# The Role of Chemistry Across Disciplines From Humanities to Life Sciences in Understanding Complexity and Emergence

**DOI:** 10.1002/anie.202523427

**Published:** 2026-03-06

**Authors:** Harald Schwalbe, Josef Wachtveitl, Alexander Heckel, Florian Buhr, Sabrina Toews, Thomas M. Schimmer

**Affiliations:** ^1^ Institute For Organic Chemistry and Chemical Biology Goethe University Frankfurt Frankfurt am Main Germany; ^2^ Center For Biomolecular Magnetic Resonance (BMRZ) Goethe‐University Frankfurt Frankfurt am Main Germany; ^3^ Instruct‐ERIC, Oxford House Oxford UK; ^4^ Physical and Theoretical Chemistry Goethe University Frankfurt Frankfurt am Main Germany; ^5^ Mainz University of Applied Science Mainz Germany

**Keywords:** complexity, emergence, chemical evolution, origins of life, genetic information

## Abstract

A group of researchers from the humanities, economics, social sciences, natural and life science developed a definition of the topics complexity and emergence that can be applied across disciplines. Here, concepts of complexity and emergence in chemistry and biochemistry are discussed, to promote a discourse between the natural and life sciences and philosophy. Although chemical research often employs reductionist strategies, the properties of molecules and their linked functions exhibit emergent properties that cannot be inferred solely from their atomic constituents. Assembly theory and the work of Manfred Eigen offer ways to quantify and predict emergence in chemistry, particularly in relation to the origins and evolution of life. This review emphasizes the chemical prerequisites for life, such as the formation of natural products, the emergence of nucleic acids that carry information, and the functional roles of proteins. From a philosophical standpoint, modern ontology provides a means of understanding reality that is both process‐based and subject‐independent. By integrating chemistry, biology and philosophy, the synopsis of this review addresses the predictive, *post facto* and historically unique aspects of complex systems, offering a conceptual framework for comprehending the emergence of molecular function and the evolution of living systems.

## Graphical abstract

This study explores the origins of life by linking prebiotic chemistry, the emergence of information‐carrying molecules such as RNA and proteins, and philosophical questions about consciousness. The study emphasizes the role of molecular evolution in the Central Dogma and provides insights into the chemical origins of biology and the basis of life's informational flow.

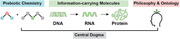

## 1 Introduction

Chemistry is concerned with the synthesis, analysis and conversion of matter. Molecules represent the constituent entities that exert function both, in natural and artificial systems. All matter on earth is constructed from atoms linked to one another through bonds to form molecules. Not all properties of molecules can be derived from the properties of atoms; in other words, molecular properties are emergent and complex. It is astonishing that in many research approaches typical in chemistry, complexity and emergence play only a minor part, if any. This situation changes at the disciplinary boundaries with physics and biology: the emergence of molecular properties and the functions associated with them, and the complexity of the microscopic and macroscopic structure of all matter are of great significance for chemical research conducted at the interface with physics and biology. Within physics, the question of how to predict the emergence of complex systems, especially of atoms after the big bang remains an open question.[1]

Currently, an assembly theory is put forward that intends to provide a framework to predict emergence [2]. The assembly theory defines objects as follows: “An object is finite, is distinguishable, persists over time and is breakable such that the set of constraints to construct it from elementary building blocks is quantifiable. (…) The more complex a given object, the less likely an identical copy can exist without selection of some information‐driven mechanism that generates such object.” These definitions and our discussion within the review show that the concept in one scientific discipline is highly influential in another discipline, as the definition quoted above is exactly in line with the concepts developed by A. Eschenmoser and M. Eigen as outlined below.

**FIGURE 1:**
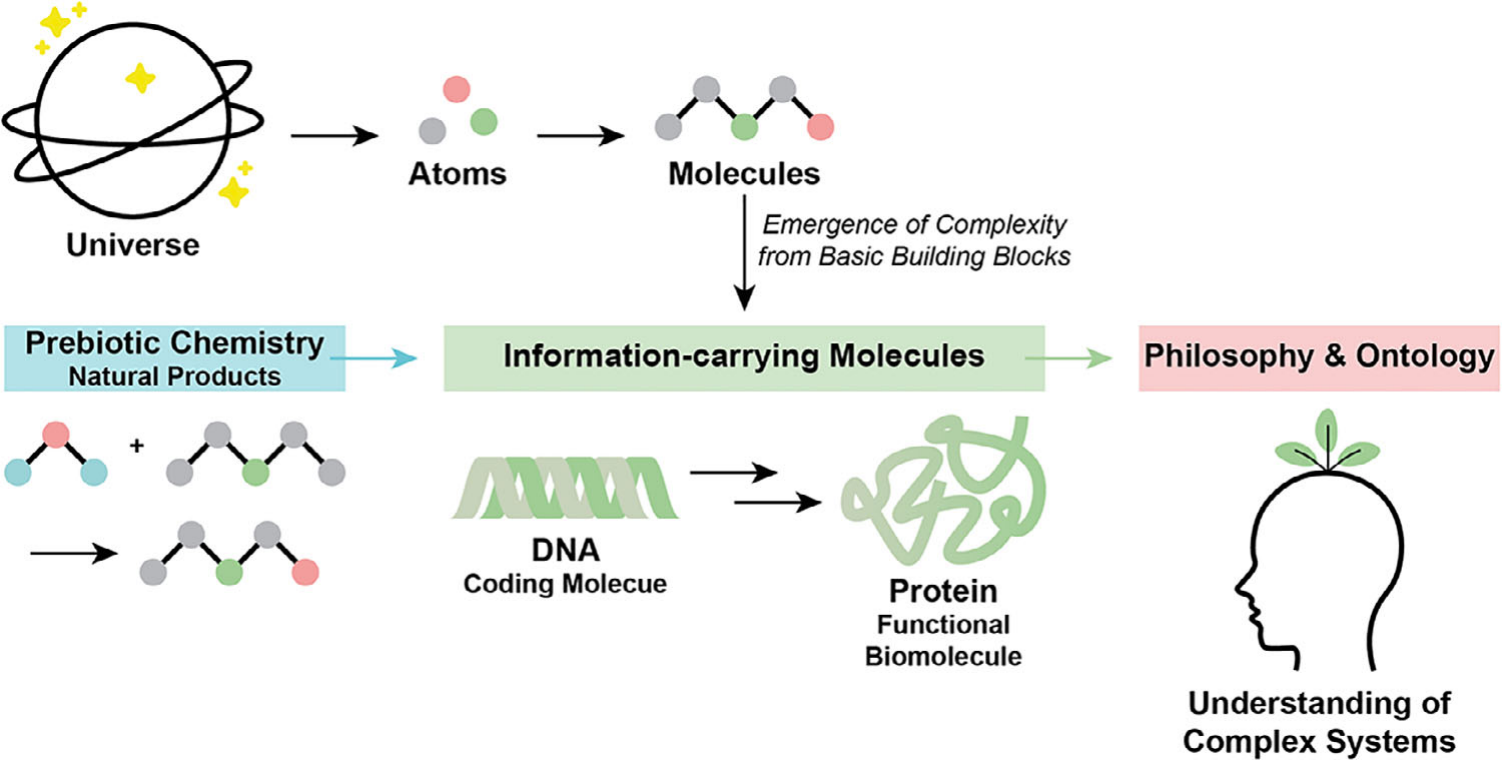
Chemistry, biology, and philosophy each contribute in distinct but complementary ways to understanding the origin of life. These contributions show how complexity arises from basic building blocks and how prebiotic chemistry creates natural products, setting the stage for life as we know it. Information‐carrying molecules, like DNA and RNA, can self‐organize and transfer information. These molecules then guide the creation of proteins, which conduct several functions inside cells, following the central dogma of molecular biology: first DNA, then RNA, then protein, then function. Combined philosophy and ontology offer a process‐based, flexible framework for understanding complex systems, connecting with chemical and biological processes. Arrows show how emergence and development happen in different areas: chemistry (blue), biology (green), and philosophy (salmon).

In this review, we attempt to delineate aspects of complexity and emergence in chemistry from a chemistry and a philosophy perspective (Figure 1). Our work has been conducted within a transdisciplinary research group joining colleagues from all scientific disciplines. Thus, we wish to emphasize here that complexity research is transdisciplinary and our definitions in chapter 2 aim at a broadly applicable definition across scientific disciplines, which requires integrating aspects from disciplines other than chemistry that are not of major importance for chemistry. By this transdisciplinary approach, we wish to document the impact of chemistry on complexity and emergence at the onset of life. Doing so, we are aware that we miss out on many important aspects within chemistry, including chemical reaction networks and systems chemistry, metabolic cycles, lipid‐based micelle formation [3, 4, 5, 6]. References to reviews on the topics might provide links to expand the discussion beyond the topics discussed in this review.

From a philosophy point of view, the investigation into the origins of life and of its evolution is part of the science of being and of that which has being. This subject matter is called ontology. Ontology is concerned with questions about basic structures and a priori characteristics of reality, and dates back to Aristotle, whose work was influenced by pre‐Socratic philosophy [7]. For Aristotle, ontology is the foundation for a “science of being in all forms” [8]. To him, ontology combines questions about those which have being and about the reality of those that are supreme and divine.

In the Early Modern Age, this connection is broken in favor of a science of being and a science of God. Since this divide, ontology has concerned itself more substantially with natural science issues. For Leibniz, ontology needs to be expanded to be a science of “that which has being and does not have being, of the thing and the nature of the thing, of the substance and the accident” (Leibniz quoted in [8]).

Not until the 20^th^ century does ontology take a new direction, which first is based on Kant but second, in terms of analytical philosophy, builds upon ontology concepts dating back to Aristotelian metaphysics. Modern ontology is often concerned with the perception of a “subject‐independent, inherently definite and yet perceptible reality” [9]. It, therefore, from “today's ‘scientified’ standpoint (i.e., with the disappearance of the transcendental or divine perspective) deals with the most fundamental structures or elements of reality as a whole” [9]. Its approaches meet the aspirations and methods of the natural sciences, because a consensus seems to have been reached that the question as to the origins and evolution of life is one which in principle can be answered, despite all the difficulties in defining the terms. From a philosophical point of view, modern ontology and natural sciences are, at least in terms of methodology, close to each other above all when “philosophical descriptions of reality […], analogously to the natural sciences, are developed and assessed as formal or at least terminologically precise theories of empirical data” [10].

For the question regarding complexity and emergence, however, in particular modern process ontology‐related approaches or those of an ontology of becoming offer parallels and potential synergies [11]. A natural science‐based understanding of the causalities of the evolution of life comprises the development of quantifiable models, which can be can be verified (at least) post factum. The natural science‐based method subdivides the generality or totality of the questions into specific sub‐questions, knowing well that difficulties frequently arise at interfaces.

In this review, we will outline chemical aspects in the context of the origins and evolution of life. The emergence of atoms after the formation of the universe preceded the development of life and the origin of species [12]. Emergence of atoms is the prerequisite for the synthesis of molecules and their conversions. More than three billion years after the formation of the universe, “larger” (polyatomic) molecules, referred to as natural products, were able to develop from simple molecules such as water, formaldehyde and ammonia. These larger natural products possess the prerequisites and properties to be able, as molecules, to perform (cellular) functions, to act as carriers of information, and hence to enable life.

A first question at the interface between astrophysics and prebiotic chemistry is the question of which chemical molecules were able to exert functions necessary to life. It was Albert Eschenmoser who raised this question concerning chemistry at the origins of life [13]. His approach was not limited to experimental work but clarified the philosophical framework for prebiotic chemical research as a key part of natural science‐based ontology (Section 3). Eschenmoser's research is mostly empirical, but through his way of defining which question to be researched, he builds a bridge to ontology and proves the fundamental empirical, constructivist approach of natural science‐based ontology. The Eschenmoser question concerning the chemistry involved in the origins of life aims at understanding which molecules evolved over three billion years [13, 14].

**FIGURE 2:**
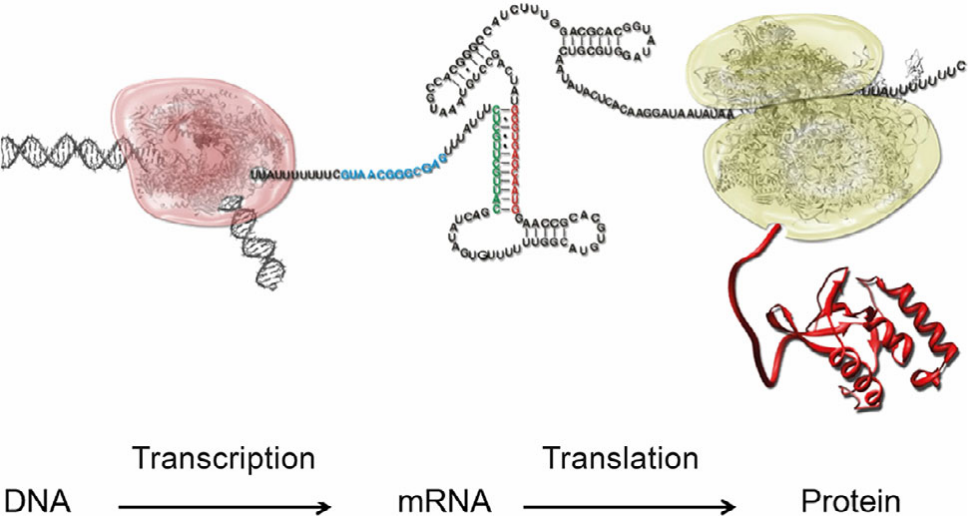
Illustration of the central dogma of molecular biology, which was formulated by Francis Crick [16]. From one of the two DNA strands, in the process of transcription a complementary mRNA (the letters shown in the middle indicate the mRNA sequence) is produced. The transcription is catalyzed by an enzyme, RNA polymerase (here shown in red). In this RNA‐polymerase‐complex the double‐stranded DNA which in the picture penetrates the complex from the left, is unwound, the newly synthesized, transcribed mRNA leaves the complex. In higher organisms, the mRNA can be additionally processed, e.g. in the process of splicing (not shown in the graphic). The transcribed mRNA contains the specification for the synthesis of proteins by the ribosome.

One of the key prerequisites of life is the undistorted transfer of information from one cell to the next (Figure 2). This transfer of information is based on the self‐organization of molecules [15]. The evolution of biological macromolecules as components of living cells was therefore subject to the selection pressure between those molecules that are capable of self‐organization. The fundamental chemical and physical properties of a self‐organizing and self‐replicating system as a basis for the origins of life have been described by Manfred Eigen [15]. The information‐carrying molecules in the cell are the nucleic acids DNA and RNA (deoxyribonucleic acid, DNA, and ribonucleic acid, RNA). The error‐free synthesis of a new DNA strand as a complementary copy of one of the two double DNA strands coming from one parent cell is the underlying principle of cellular information transfer. The participating DNA molecules are, for this reason, referred to as genotypic molecules. The chemical properties of the genotypic molecules are discussed in Section 4. However, evolution does not end with the transfer of cellular information – the transfer of information does not yet itself constitute life. It is not an end, rather, the information which is saved in readable form in the sequence of the DNA building blocks codes, in today's organisms, for diverse biological functions which are performed by proteins, above all by enzymes. Proteins, as a third essential category of biomacromolecules alongside the nucleic acids DNA and RNA, permit the vast variety of cellular functions and hence the greater diversification of more highly evolved organisms and their specific organs. Proteins are consequently called phenotypic molecules. The information transfer at the beginnings of life therefore had to be expanded from the self‐replication capabilities of nucleic acids to transfer of information into function, exerted by proteins.

With his central dogma of molecular biology, Francis Crick created the theoretical framework for describing the process of synthesis of biomacromolecules, particularly regarding their directionality [16]. The process of cellular information transfer requires the error‐free synthesis of fundamentally different categories of biomacromolecules, namely nucleic acids and proteins. Within the cell, these syntheses take place on two macromolecular complexes which are themselves assembled during transcription and translation. Polymerases catalyze the transcription of one of the two DNA strands into a new DNA strand during replication, or into an RNA strand during transcription [17]. Ribosomes bring about the synthesis of proteins in accordance with the information stored in the chemical sequence of RNA building blocks [18]. The information and translation specification, referred to as the genetic code, underlying these syntheses, was deciphered by Nirenberg and Khorana [19, 20]. The genetic code is universally valid for all living organisms.

The sequence of an mRNA, i.e., the order of the RNA building blocks, provides a code for the sequential order in which the amino acids are arranged in the protein. Proteins are synthesized on the ribosome (here shown in green, the ribosome consists of two sub‐units). The mRNA sequence is recognized and decoded by tRNA, a hybrid molecule of RNA molecules and an amino acid. The newly synthesized protein chain carrying hundreds of amino acids leaves the ribosome. The synthesis both of the mRNA and of the protein is followed by a process known as folding, in which RNA and proteins take on their 3D shape (3D structure). This 3D structure provides the basis for the function of the biomacromolecule.

This review intends to build a bridge between natural science phenomena and the “Mainstreams of contemporary philosophy”, to quote a standard reference by Wolfgang Stegmüller [21]. In Volume III of his monograph, Stegmüller describes the theory of evolution by Eigen and thus permits an exchange between the philosophical and the chemical, biochemical and biological aspects of evolutionary theory. Stegmüller points out that one “tends, as a philosopher”, to split up “the problem of the origins of life into two sub‐questions. Thus, in a first step, the question ‘What actually is evolution?’, would need to be answered, whilst in a second step, one would need to supply a sound theory explaining the evolutionary process. But it does not work like that. An important factor in the appropriation of modern evolutionary theories, such as that of Eigen, might be recognizing *why this approach cannot be followed*. The concept of evolution is much too complex and comprises far too many different types of individual phenomena, processes and involved laws to be able to immediately begin the explication of this concept. Rather, what is important is to split up the problem of evolution into a series of individual questions that can be worded scientifically, to arrange these questions in the correct order, and in each case to *select the right starting point* for solving the problem” [21].

Further important aspects of a philosophical theory of complexity have been provided by Hans Poser [22]. In his third chapter “Evolution as a scheme of interpretation” he quotes Kant: “It is […] certain that we will not be able to even adequately get to know the organized beings and their inner possibilities according to purely mechanical principles of nature, much less be able to explain them to ourselves; and indeed so certain, that one can say boldly that it is absurd for humans to even make such an attempt, or to hope that one day another Newton might arise who could enable us to understand even the creation of a blade of grass according to the laws of nature not intentionally ordered” [22].

Given that understanding the inherent chemical properties of molecules allows us to describe evolution of a blade of grass, it is the intention in this article that systems complexity and emergence are important concepts not only in philosophy but also in chemistry. If so, then a complexity theory ought to be able to distinguish between (i) a predictive capability, where applicable specifying a probability, (ii) a *post factum* inherently logical, but not (unequivocally) predicable evolution of a system and of (iii) an in principle non‐repeatable evolution, in other words of an essentially historical process. It is not the historical outcome of the process, i.e. the result of the evolution that is in principle predicable, but rather the evolution's process *type*. The use of the term “process type” comes from Poser [23].

## 2 Definition of Complex Systems and Their Evolution

The terms “complexity”, “complex systems” and “evolution of complex systems” are essential for many scientific disciplines. These range from natural sciences and medicine to the humanities and social sciences. For these terms to be useful across many disciplines the existence of a uniform concept and definition of complexity is essentiell. Any transdisciplinary attempt using the term complexity fails if it is not possible to agree at least on a set of basic elements of a complex system and its evolution in time. Transdisciplinary research of complexity requires concepts that share some level of uniform definition, which may have to be adopted to the specific semantics of a sub‐discipline. Hereafter, we introduce thirteen points that define the characteristics of complex systems and describe their time evolution. These definitions should be valid and applicable for systems beyond single disciplines.

1. Most complex systems are dynamic and evolve in time. In a strict sense, their evolution is irreversible and not reproducible. The deviation from exact reproducibility can, however, be very small.
2. Living complex systems are open systems. Openness fundamentally implies that the complex system can be separated from its environment. The environment itself can disintegrate into further (complex) systems. Exchange between the complex (inner) system and its environment is possible in many ways; an inevitable exchange exists in the form of energy exchange. The complexity of the environment can often be greater than the complexity of the system.
3. A complex system consists of particles/members/elements (hereafter called elements). The elements have properties that define the properties of the complex system. The number of elements is sufficiently large so that both, stochastic fluctuations and directional flux of the properties associated with the properties of the molecules can be observed.
4. The elements of a complex system interact with each other. The interactions can be described and depend on the configuration of the system (number of elements, position of the elements in space). The sum of the interactions can but does not have to be linear or additive.
5. The configuration of a system can be described at any time of its evolution.
6. A complex system has characteristic properties. These properties can be intensive or extensive, i.e. (linearly) dependent or independent of the size of the system. A complex system can have and/or execute a function. One function associated with enzymes as part of a complex system encompassing reactants within a (partially) closed system is to catalyze a reaction. Function can exist through the interaction of a partially closed system with another, external system. The transition from a simple system to a complex system is usually gradual.
7. The elements of a complex system can be arranged in different hierarchical orders. In general, there is exchange between these hierarchical levels; the exchange can be reversible or irreversible, directed or balanced. Often, but not necessarily, complexity in dynamic complex systems at higher levels evolves from the properties of the elements of lower levels. In complex systems which are time‐independent, complexity may be a static property of higher orders.
8. In principle, the evolution of a complex system is driven by a nearly constant energy input. This constant energy input causes systems to change from a state of lower complexity to a state of higher complexity. The extent of complexity can be described or quantified within the theory of complex systems.
9. The necessities of the functions of a complex system can be used to select one configuration from other configurations of this system (Darwinian Evolution).
10. In principle, the evolution of non‐complex systems can be described. Individual configurations of the system can be predicted with a very low probability but are predictable in principle. Evolution of complex systems comes up against points or periods of criticality; at these points of criticality the properties of the system change in a way that is not predictable for fundamental reasons, in principle. The unpredictability of the new state of the system does, however, not imply that the new state is not compatible with the laws of evolution of the system.
11. In the transition of both, static and dynamic complex systems at points or periods of criticality, complex systems emerge. This emergence can consist of a transition of all or only single elements of the system.
12. Phases of emergence alternate with phases of homeostasis. Complex systems are distinguishable from chaotic systems. The characteristic of chaotic systems is their sensitive reaction to minimal stochastic modulation of the initial conditions. Complex systems can evolve to homeostasis from basically very different initial conditions. Complex systems are characterized by self‐organization. As part of self‐organization, a dynamic complex system can evolve into a (metastable) state. It remains open, whether this evolution is linear or cyclic.
13. In social systems, complexity is defined as the degree of multifariousness, interconnectedness and consequential weight of the decision‐making field of a social system. The more complex a specific situation is, the more relevant options for action of decision and their criteria arise as to how complex dynamic systems can be organized. In principle, decisions are possible in phases of homeostasis, but their effects are unpredictable at points of criticality.

## 3 Etiology of Potentially Prebiotic Molecules Important for Biological Function

**FIGURE 3:**
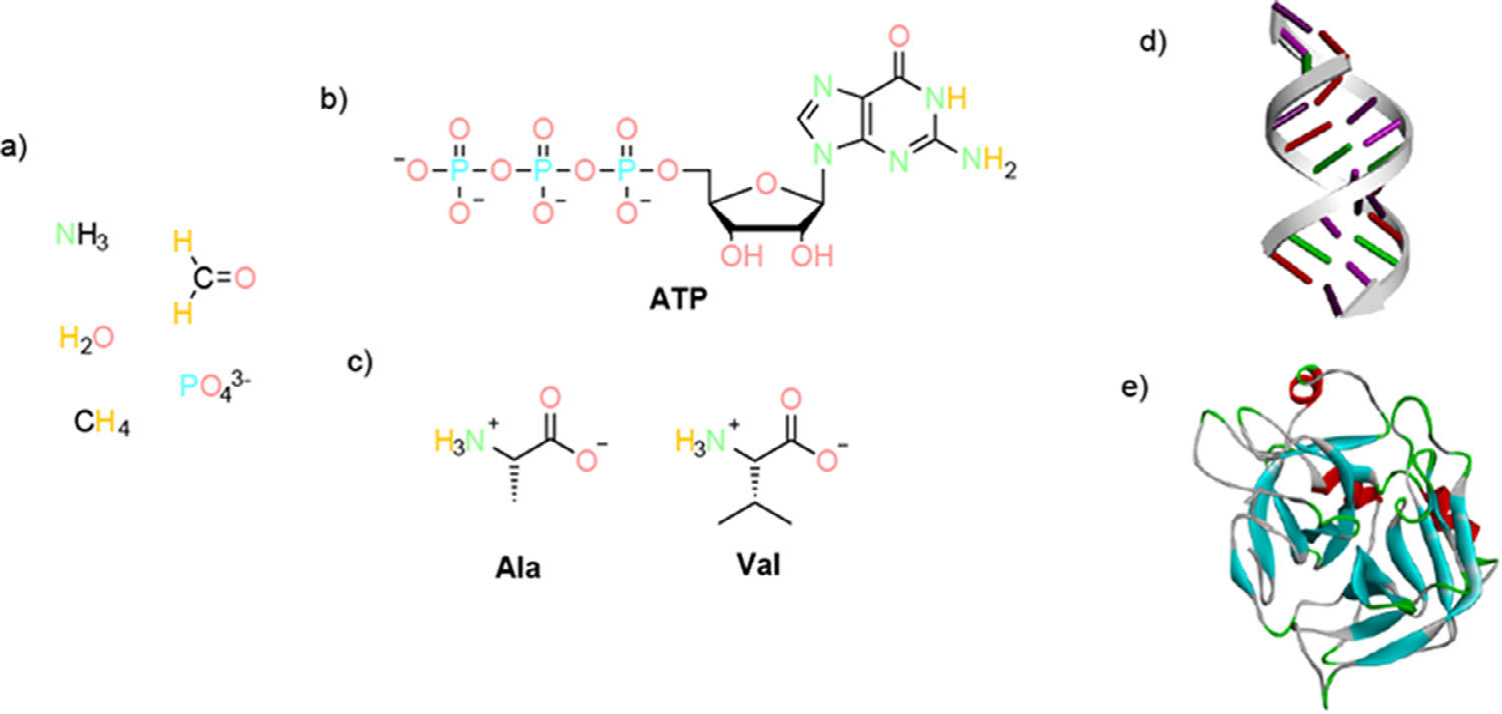
Evolution of chemical molecules. (a) Simple molecules that were found in stars and stardust. In the period of chemical evolution, prebiotic molecules developed: (b) prebiotic, RNA building blocks activated for reaction using the example of the RNA building block adenosine triphosphate (ATP) and (c) prebiotic amino acids alanine (Ala) and valine (Val). (d) RNA double helix: two complementary RNA strands can form an RNA double helix, which has similar but not identical properties compared with a DNA double helix. (e) 3D structure of a protein.

When life evolved, there was a period during which initial molecules must have formed which then served as building materials for the first viable cells (Figure 3). Below, we outline the methodological and philosophical framework of the etiology of such potentially prebiotic compounds. This framework was developed by Albert Eschenmoser.

The term “etiology” describes science of causes and origins of things. In the context of the origins of life, therefore, the question arises as to the origin of the molecules which could be deemed the “last common precursors” of all biological cells and organisms. The development of these “prebiotic compounds” began four billion years ago, but we know just as little about the precise timeframe over which these compounds emerged as we do about the chemical properties of the environment (in an aqueous medium, on inorganic surfaces, or in the gas phase) in which they did so. There seems to be a consensus, however, that the first single‐celled organisms emerged approximately 3.8 billion years ago, the first mammals 200 million years ago, *Homo sapiens* 200.000 years ago, and the ability to cultivate land 10,000 years ago. During evolution, therefore, we observe an acceleration of the evolutionary steps.

The emergence of prebiotic molecules was preceded by the formation of chemical elements and atoms: hydrogen (H_2_) and helium (He) were generated during the big bang. All other chemical elements originated in stars. The prebiotic molecules water (H_2_O), ammonia (NH_3_), methane (CH_4_), formaldehyde (H_2_C═O) and phosphate (PO_4_^3−^) emerged in specific gas mists and may be sufficiently stable under specific conditions.

To get closer to the question of the etiology of potentially prebiotic biomolecular molecules, one cannot rely on solid historical facts, not even in the sense of a still‐existent relict of these molecules in “primitive” organisms. Instead, by way of fundamentally accessing research into this prebiotic chemistry, hypotheses need to be formulated about possible molecular scenarios. These hypotheses are tested for plausibility using experiments. The detection of potential molecules, in other words the molecular players in the phase of evolution of prebiotic molecules, is designed to answer the question: could these molecules come into being? Over and above this, the investigation into their evolutionary selection is necessary, so: why were they able to survive, with respect to the evolution of life, under varying selection scenarios?

Both, the conditions for the synthesis of prebiotic molecules and the function performed by the prebiotic molecules are subject to selection pressure. Nevertheless, the research into both questions differs in a crucial point: the question about the etiology of the first prebiotic molecules is historical. The conditions from that time can no longer be reproduced, it is impossible to prove what the “winning” boundary conditions were. Consequently, they remain speculative.

Function that is performed by one or more prebiotic, but still existing, molecules is, however, time‐invariant. It can thus be precisely investigated whether molecules, in whatever way they have been selected, *can* actually perform their function. The stability under given environmental conditions of possible prebiotic molecules is a thermodynamic property, which is constant and unchanging. Maybe other molecules have also developed. These alternative prebiotic molecules or molecular systems may, in the course of evolution, have been chemically stable under given environmental conditions, but could not have been necessary any longer or became instable during the evolution, for example by changing from an anaerobic to an aerobic environment. In this context, the sometimes futuristic sounding question about life in the universe is of interest, since it may be that other molecules (under different environmental conditions) could have evolved. Here, a distinction – desirable in ontology – between things, processes and results therefore does not seem possible, because even with the chemistry of potential prebiotic molecules, we have a case of “things” merging with the process of their development. “The models do not deal with objects and their properties but with *processes*. Ilya Prigogine and Manfred Eigen spoke of a transition from being to becoming, and Whitehead already proposed a process ontology” [22].

The legendary first experiments in prebiotic chemistry date back to Miller and Oró [24, 25]. Their experiments provided chemical evidence regarding which prebiotic molecules can form. (Non‐chiral) α‐amino acids can be formed from the above‐named “more primitive” precursor molecules hydrogen (H_2_), methane (CH_4_), ammonia (NH_3_) and water (H_2_O) [24, 26]. The nucleobase adenine as a precursor molecule of the nucleic acid building blocks can be formed from ammonium hydroxide (NH_4_OH) and hydrogen cyanide (HCN) [25]. Sugars can be formed from glycolaldehyde phosphate [13]. These molecules, α‐amino acids, and the linking of nucleobases, sugar units and phosphate(s) constitute the monomeric units which, by means of polymerization reactions, form the biomacromolecules essential to all cells, such as proteins and nucleic acids (DNA and RNA) [27, 28]. They are generated in these prebiotic experiments under a large number of conditions as soon as energy and the above‐named precursor molecules are present. Interestingly, these monomeric units are also found extraterrestrially [29, 30, 31].

As interesting as these experiments are, they are, however, far away from explaining the next steps, which lead to life, namely in what way, potentially, life has emerged from prebiotic molecules through self‐organization. Prebiotic chemistry, even today, is by no means in a position to produce a theoretical model of this complexity nor to simulate it experimentally. The latter in particular is exciting: experimental simulation, which constitutes (chemical) life would represent impressive inductive proof of understanding. In this context we refer to Bernal's bonmot which was quoted by Eschenmoser: “If life once made itself, it must not be too difficult to make it again.” [13] In prebiotic chemistry, questions about the “Why” are an essential component: with these questions which are otherwise prohibited in the natural sciences due to being of teleological nature, in prebiotic chemistry the issue is why e.g. the nucleobase moieties in nucleic acids are linked via phosphate groups instead of alternative atomic bridging units such as sulfates, silicates or arsenates [32]. Comparative experiments with the selected molecules and possible chemical alternatives address both the question of whether, under given conditions, a compound can in principle be generated and serve as a starting point for answering the selection problem. As stated above, investigations into the function of such candidate molecules are time‐invariant. If, therefore, one of the alternative molecules cannot perform a desired function, then although it could in principle have emerged under prebiotic conditions, owing to a lack of function it would not have been able to stand up to the evolutionary selection pressure. It should also be pointed out that while evolution as defined by Darwin is continuous, it does not have to be linear. Certain molecules could, at certain points in time, have contributed during evolution, but might have died out under the changing conditions because improved versions took over the function or indeed completely new players hijacked the function.

To conclude, it should be mentioned here that prebiotic chemistry is in no way a self‐contained scientific field. The questions being dealt with are excellent at providing information about the fundamental reactivity of chemical compounds and thereby extend far beyond prebiotic chemistry into general contemporary synthesis chemistry [33, 34]. This much can be said: the monomeric building blocks of all central information‐ and function‐carrying biomacromolecules in organisms, the genotypic DNAs, the phenotypic proteins and the RNAs which combine the geno‐ and phenotypic properties were able to emerge under prebiotic conditions and have prevailed over similarly possible chemical compounds.

## 4 Nucleic Acids DNA and RNA as Carriers of Cellular Information

On this basis, we shall discuss below how genetic information can be replicated from the chemical structure of the nucleic acids [12, 35, 36, 37, 38, 39, 40, 41, 42, 43, 44, 45, 46, 47].

**FIGURE 4:**
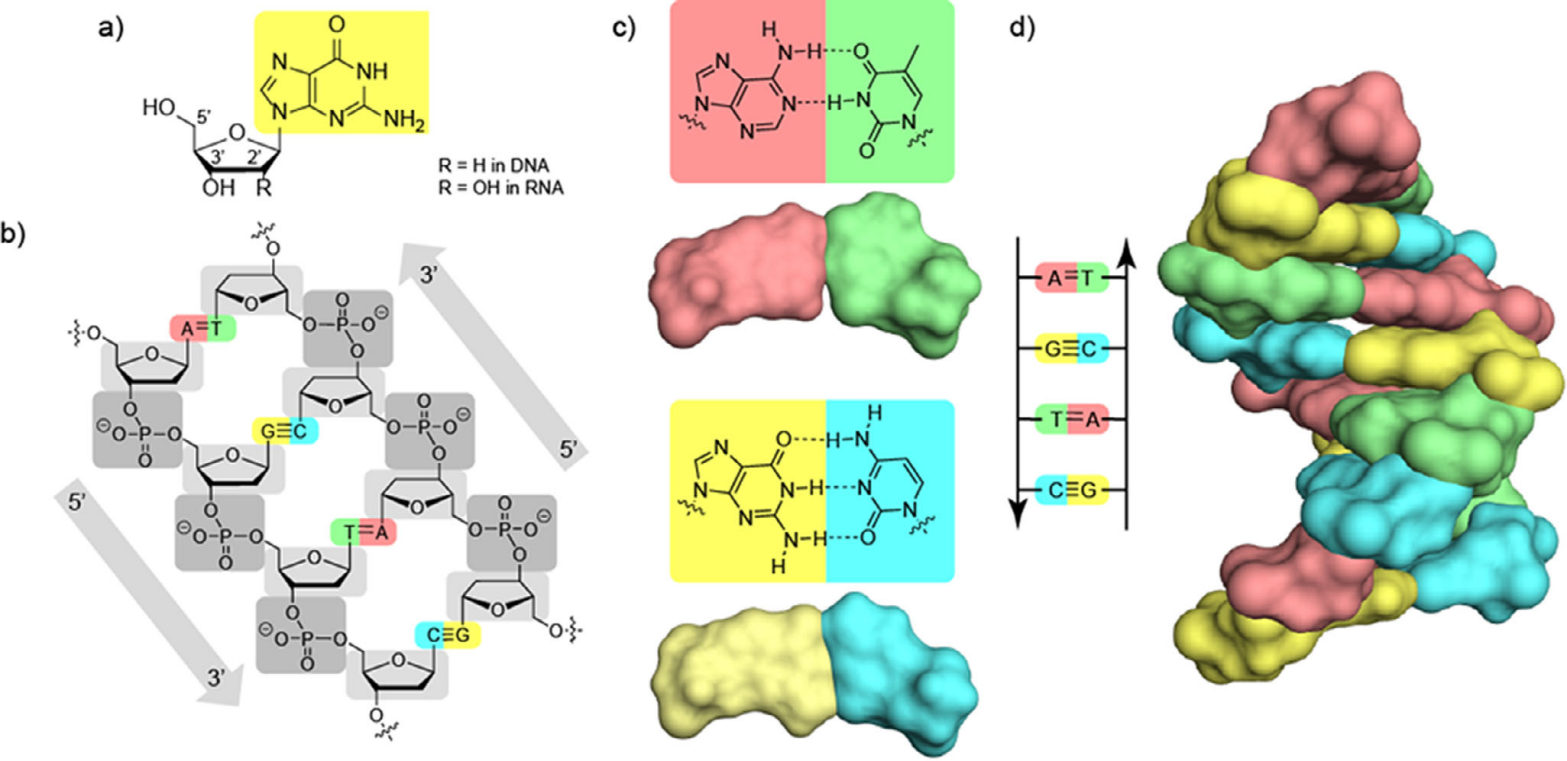
(a) The monomeric building block guanosine (R═OH) is one of the four basic building blocks of RNA, the variant deoxyguanosine (R═H), occurs in natural DNA. (b) One DNA strand consists of a sequence of consistently the same deoxyribose (light gray) and phosphoric acid diester units (dark gray) and has a direction (arrow). Each deoxyribose unit can carry a variable, information‐carrying nucleobase, which may be either A (adenine, red), G (guanine, yellow), T (thymine, green) or C (cytosine, blue). (c) Via hydrogen bonds (dotted), nucleobases can interact with each other. The pairing from A═T and G═C stands out for the fact that they have the same shape (in each case, see the depiction of the formula and the surface). (d) From these base pairs a DNA double helix – similar to a ladder but rotated helically—is generated, through stacking.

Nucleic acids are long, linear – i.e. unbranched – molecules built according to a very simple principle. Nucleic acids are copolymers, generated from stringing together of four building blocks. It is important to note here that a nucleic acid, similar to a vector or multi‐digit numbers, has a direction – i.e. a beginning and an end. In order to understand how a nucleic acid is capable, as a molecule, of being an information store, we must first look at the structure more closely (Figure 4). The key to understanding the information‐carrying principle of nucleic acids comes about from an understanding of the interactions of two nucleobases (colored elements in Figure 4). There are two forces which must be considered here: hydrophobic interactions and hydrogen bonds. The hydrophobic interactions ensure that one can stack nucleobases on top of one another. This will immediately become important for the understanding of the structure of the nucleic acids, but this interaction is unspecific and essentially the same for all nucleobases. Only when one also factors in the interactions via the hydrogen bonds is the information‐carrying principle revealed in its full elegance. Simple theoretical considerations which can, however, be proved experimentally, show that in each case two of the four nucleobases, directed by the interaction principle of the hydrogen bonds, can find each other by themselves, that is by self‐organization in 28 ways. Now these aggregates, in a similar way to the plugs and sockets that we are familiar with, are not fixed immediately. Precisely one of these pairs contains the maximum possible number of three hydrogen bonds – the pair composed of G═C (hydrogen bonds are indicated with lines in this notation) – whilst all others have fewer. This makes this pair particularly stable. If one now compares all other 27 possible pairs with this one, then among them there is precisely only one other one – the pair composed of A═T with two hydrogen bonds – which has the same shape externally (!) (Figure 4c). Each of both of these “Watson‐Crick base pairs” – named after the two Nobel Prize winners who clarified the structure of DNA – consists of a shorter pyrimidine nucleobase and a longer purine nucleobase, which when combined have the same length, width and height. Together with the previously described vertical “stackability” of these flat base pair slices, the construction principle is revealed, which is simultaneously the key to understanding the information processing: every nucleobase in a nucleic acid strand has its matching, “complementary” nucleobase and forms a base pair. Owing to the formal principle, these may in turn be connected via their own deoxyribose and phosphoric acid units to a complementary strand (Figure 4b) and the entire construct is held together via the hydrogen bonds in the flat base pair slices and – perpendicular to this – via the hydrophobic interactions. Owing to its phosphodiester groups with a negative charge, the backbone of each of the two strands is hydrophilic and hence ideally suited for interaction with the surrounding water, whilst the stated hydrophobic base pairs – by their nature interactive with one another – come to rest internally in this structure and hence shielded from the water. The image of a ladder presents itself (Figure 4d), in which the internal hydrophobic base pairs acquire the role of the rungs and in which both of the “hydrophilic bars” are formed by the deoxyribose phosphodiester backbone – and with a ladder, too, all rungs must be equally wide. In DNA only, the rungs consist of an A═T, T═A, G═C or C═G base pair and therefore can transmit information. For completeness's sake, it should be mentioned that such a double strand of DNA, unlike a ladder, is twisted in a spiral manner (helically) along the axis (Figure 4d). This is the often‐mentioned DNA double helix. It should also be mentioned that the directions of strand and counterstrand in this helix are opposing (antiparallel). Other arrangements are also conceivable, but only the one just described has this kind of regularly and is therefore ideal as a basis for an information‐carrying and information‐processing system. RNA double helixes are very similar to the DNA double helixes, in particular, the structural principles are very similar. The monomeric building blocks of the DNA and RNA nucleic acids are chiral, and the DNA and RNA helices likewise.

## 5 The Structure of Proteins as Functional Biomacromolecules

**FIGURE 5:**
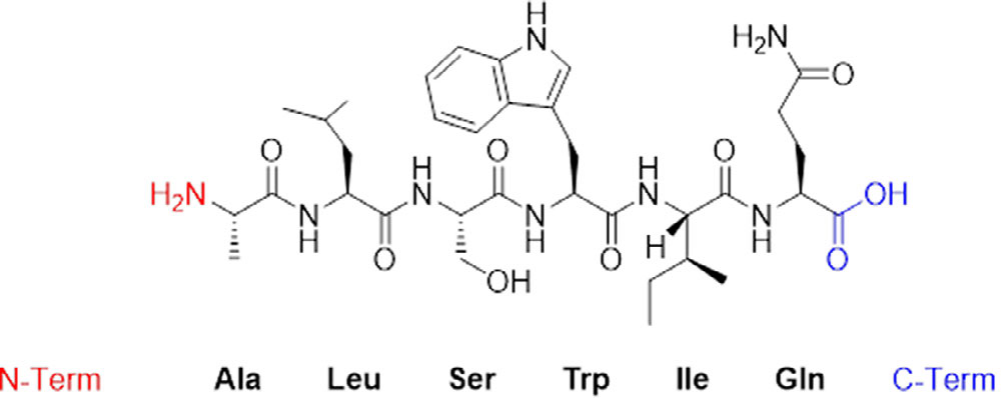
Polypeptide chain made from the six L‐amino acids alanine (Ala, A), leucine (Leu, L), serine (Ser, S), tryptophan (Trp, W), isoleucine (Ile, I) and glutamine (Gln, Q). On the left‐hand side is the N‐terminal end with an amino group (NH_2_ group, red), on the right‐hand side is the C‐terminal end with a carboxylic acid group (COOH group, blue). A line represents a chemical bond (a single bond), two parallel lines a double bond. Wedges and dashed lines indicate the chirality on the tetrahedral carbon atom.

Like nucleic acids, proteins are polymers. A protein is constructed from amino acids as monomeric building blocks (Figure 5). Simple amino acids were detected as prebiotic molecules (Section 3). Essentially, there are 20 different proteinogenic amino acids. Owing to the existence of 20 different amino acids, a number that is large compared to the four different nucleotides, there is a very large number of possible peptide sequences, precisely: for a peptide of n amino acids, 20^n^ different sequences (Table 1).

**TABLE 1:** The number of possible sequences is very large compared with the number of proteins found in nature [48].

Name	Number of amino acids n	20^n^ Number of possible sequences
Dipeptide	2	400
Tripeptide	3	8,000
Tetrapeptide	4	160,000
Pentapeptide	5	3,200,000

The 20 different amino acids are annotated with a letter (I, L, V, A etc.) or with three letters (Ile, Leu, Val, Ala etc.).

**FIGURE 6:**
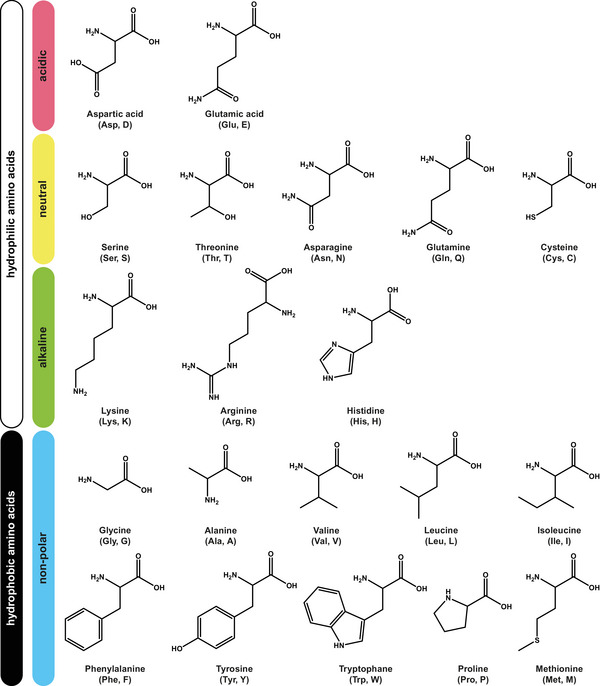
Properties of amino acids which originate from the different substituents R in the side chains of the amino acids.

Owing to their substituents R (or moieties R), the amino acids have different properties (Figure 6). (The atoms of) amino acids interact with each other. Owing to their differing properties, as indicated in Figure 6, amino acids can interact with each other differently. Put very simply, two essential interactions may be distinguished here: hydrophilic or polar (P) interactions, especially between charged amino acid moieties, and hydrophobic (H) interactions between uncharged, (poorly) water‐soluble amino acid moieties. The twenty different side chains interact in an attractive or repulsive manner, based on charge‐charge interactions, interactions with solvent water that differ between the surface of the protein and its interior, hydrophobic interactions as a driving force for compaction of the interior of the protein, driving the early steps of folding. A detailed discussion of these driving forces is given in Holehouse and Pappu [49].

**FIGURE 7:**
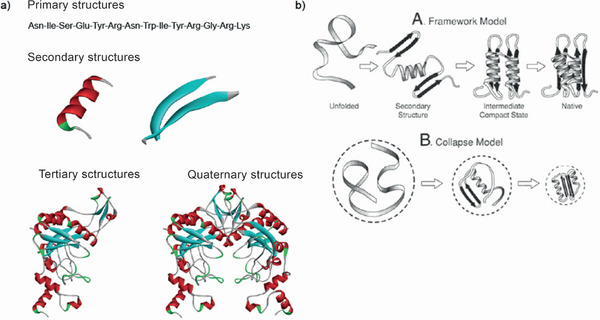
(a) The hierarchies of the protein structure are subdivided into the sequence of amino acids (also termed primary structure), different secondary structures (α‐helix and β‐pleated sheet); the arrangement of the secondary structures to one another (described as tertiary structure). Ultimately, two proteins can interact with one another, and form a protein‐protein complex (termed quaternary structure). (b) Models of how the tertiary structure of a protein can form. The starting point of the structure formation of proteins (known as protein folding) is the unfolded state, which can adopt the folded state (native state) via different intermediate stages (“secondary structure”, “intermediate compact state”). Figure adapted from Dill et al. [52].

Owing to the interaction between individual atoms and atom groupings of the amino acids in proteins, these assume different conformations (“structures”) in the 3D space (Figure 7). The systematic structural analysis of proteins was established by Linus Pauling (Nobel Prize for Chemistry 1954) [50, 51].

## 6 The Genetic Code as Universal Basis for Cellular Information Transfer

In today's organisms, nucleic acids, especially DNA, are carriers of genetic information. In viruses, RNAs can be the carriers of genetic information. Owing to the complementarity of DNA building blocks in strands arranged in antiparallel, as described in Section 4, and owing to the chemical stability of DNA (even today, it is possible to determine the DNA sequence of Neanderthals), DNA as a long‐term information store possesses a crucial selection advantage, for example compared with RNA. With respect to the diverse requirements placed on biomacromolecules in living organisms, however, DNA molecules are limited in their function. Not least the structural rigidity of DNA is one of the reasons why, even now, only very few DNA molecules are known to possess enzymatic function.

Essentially, DNA molecules form rod‐like structures. From the analysis of the function in contemporary organisms, which is time‐invariant, it seems virtually impossible that DNA molecules could have promoted and catalyzed their own replication or were involved in the origin of life. In other words: how a parent DNA can act as a template and be copied *in an exact manner* as postulated by Haldane (see reference 35), is explained by Watson and Crick through the structure of the DNA double helix [53]. For the exact synthesis of the daughter strand, the linking of the complementary monomeric DNA building blocks, the integration of the monomeric building blocks must be catalyzed.

This situation produces a “chicken and egg” problem: how are the functional phenotypic molecules generated that catalyze the replication of the genotypic molecules if, at the transition from the inanimate to the animate, precisely those phenotypic molecules did not exist? In all today's organisms, proteins take over these catalytic functions. Because of the diversity of their 20 amino acids, they can form 3D, fissured structures. The molecular biological “chicken and egg” problem is therefore: What came first: DNA or proteins? One possible experimental clue, and one that is by now accepted as very probable, toward answering (“neither of them”) this question is provided by the work of Tom Cech and Sydney Altman, which was honored with the Nobel Prize in 1989 [54, 55]. The two scientists were able to prove for the first time for two independent systems that RNA molecules, unlike DNA molecules, display both genotypic and phenotypic properties. RNA molecules can perform enzymatic functions. Such RNAs are termed ribozymes (from components of the words **ribo**nucleic acids and en**zyme**). In the following, we will discuss aspects of complexity and convergence in the context of RNA chemistry and biology. Doing so, we do not wish to imply that the question of the transition from the innate to the animate has been ultimately solved. Currently discussed hypotheses include assembling systems capable of self‐replication, co‐evolution of RNA‐peptide systems, peptide amyloids as primordial functional polymers, as well as stressing the importance of inorganic mineral‐based protocells to achieve prebiotic fitness [56, 57, 58, 59, 60, 61].

**FIGURE 8:**
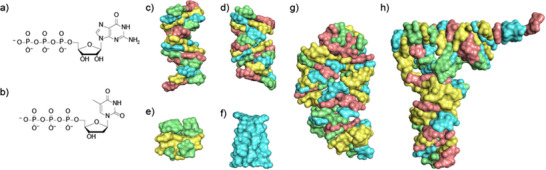
(a) RNA and (b) DNA triphosphates. DNA structures: (c) and (d) DNA double helix (B and A shape), (e) G quadruplex, (f) i‐motif; RNA structures: (g) RNA double helix (A shape), and (h) tRNA.

The need for existence of molecules, at the origins of life, with autocatalytic properties, is discussed by T. Cech in his Nobel Prize speech as follows: “A living cell requires thousands of different chemical reactions to utilize energy, move, grow, respond to external stimuli and reproduce itself. While these reactions take place spontaneously, they rarely proceed at a rate fast enough for life. Enzymes, biological catalysts found in all cells, greatly accelerate the rates of these chemical reactions and impart on them extraordinary specificity” [54]. The proof that RNA molecules, which are either found in organisms or can be developed in the laboratory by means of what are known as in vitro selection experiments, can catalyze the synthesis of monomeric RNA building blocks, their own replication and also the ligation of two RNA strands, as a time‐invariant function, corroborates experimentally the hypothesis uttered much earlier by Woese, Crick and Orgel of an RNA world which must have preceded a world in which life is based on function linked to DNAs, RNAs and proteins [62, 63, 64, 65, 66, 67, 68, 69, 70, 71, 72, 73]. The functional diversity of RNA is linked to the fact that RNAs, in a similar way to proteins and in contrast to DNA, can assume 3D structures, which can catalyze a large number of different reactions (Figure 8). Interestingly, a DNA double helix, which takes a different form from an RNA double helix, cannot catalyze the attachment of further dNTPs. The catalytic capabilities of RNA molecules, with only four different nucleobases may be limited compared with the functional diversity of proteins, but relicts of RNA‐based catalysis are ubiquitous in today's living cells. For example, the essential step of peptide bond formation in the ribosome is catalyzed by RNA molecules. It is now therefore viewed as extremely likely that an “RNA‐only world” preceded the coexistence of a system consisting of RNA, DNA and proteins. In this context, a “DNA‐only world” is ruled out because of the lack of autocatalytic properties of DNA, and that a “protein‐only world” can be ruled out because of the lack of a mechanism for self‐replication. The self‐complementary nature of the nucleobases necessary for error‐free self‐replication is not found in the 20 contemporary amino acids and this is still not the case if there had been fewer than 20 amino acids at the beginning. The precise complementarity of the nucleobases in the nucleic acids does not have a corresponding molecular basis within the amino acids.

In today's organisms one finds proteins, DNAs and RNAs. Alongside the RNAs capable of replication in an “RNA‐only world”, during evolution DNAs and proteins must have been added. The likely evolution would have been from an “RNA‐only world” via an “RNA‐protein world” into a “DNA‐RNA‐protein world”. It then requires the synthesis steps recorded in the central dogma of molecular biology, namely that of the exact reproduction of the DNA during replication and that of the conversion of the DNA sequence information into protein sequence information during the translation. After repetitive synthesis steps the biomacromolecules undergo a folding process to aquire their three‐dimensional structure. Principle aspects of protein folding, in the context of complexity and emergence, are reported on in Sections 8 and 9.

In today's organisms, the process of transfer of cellular information is not a direct process from genotypic DNAs to phenotypic proteins, rather, first a copy of the DNA strand, a complementary RNA, is produced in the process of transcription. From a chemical perspective, transcription from DNA to RNA is much simpler than the translation from nucleic acids to proteins, because DNA and RNA are very similar and capable of self‐organization. In cells, RNA is four times as common as DNA. The enzyme ribonucleotide reductase (RNR) brings about the reduction of the RNA OH group to the DNA H atom [74]. Thus, the evolution of a primitive form of the enzyme RNR during the transition from the “RNA‐protein world” to the “DNA‐RNA‐protein world” is necessary to produce monomeric DNA building blocks from monomeric RNA building blocks. If DNA building blocks are present and in a chemically activated form, then DNA‐RNA, but also RNA‐DNA, transcription is completely comprehensible. It is based on the complementarity, described in Section 4, of the nucleobases G and C and of A and T for DNA or U for RNA.

On the other hand, the development of a system comprising four different RNA building blocks into a system with 20 different amino acids required the chemical evolution of a completely new translation system and the evolution of a genetic code. In some way, RNA building blocks must be connected to amino acids in a precise manner, conserving the information encoded in DNAs. Nature's rules for this information transfer are called the genetic code. Nirenberg and Khorana experimentally deciphered the genetic code in 1961–1962, and in 1968 Crick and Orgel held the discussion about the fundamental requirements for the evolution of the genetic code.

Thus, there must, at the origins of life, have been a transition to a system which contained the different categories of biomacromolecules. To this day, this evolutionary transition is not fully understood, as Orgel stated as early as in 1968: “We have seen that ‘organisms’ without nucleic acids would lack the means of achieving genetic continuity, while organisms without proteins would be severely limited in their ability to use the chemicals in their environment. The difficulties associated with theories of the direct evolution of life as we now know it are of a quite different kind. While the practicability of organisms using both nucleic acids and protein is not in doubt, we do not understand how they could have evolved. In particular, we do not understand the origin of the genetic code which provides the critical connection between the genetic and the functional apparatus of the cell” [64]. Even more than 50 years later, the puzzle has not been solved.

The fundamental requirements concerning the development of a self‐replicating system made from RNA and proteins consist of the following aspects:

1. An amino acid must be specifically bound to the self‐replicating RNA; an RNA‐amino acid‐containing hybrid molecule has to form. This specificity, that precisely one amino acid is always bound to an RNA made up of, as the case may be, several RNA building blocks, constitutes the prerequisite for the precisely transferred RNA sequence information being converted into a successive integration of what is always the same amino acid into the growing polypeptide chain.
2. From the above point, two distinctly different functional roles are generated for the two RNA molecules. The self‐replicating RNA molecule contains the message, and the contemporary form of this functional module is named messenger RNA (mRNA). The hybrid molecule made from RNA and amino acid transfers this information to a newly synthesized polypeptide and is called transfer RNA (tRNA).
3. For the synthesis of a dipeptide, two RNA‐amino acid‐hybrid molecules, two tRNA molecules, must come together through self‐organization. This coming together, this self‐organization of two tRNA molecules, is brought about by a template effect of the mRNA (Figure 11, Equation 5). Two directly adjacent codons on the mRNA provide the blueprint for the assembly of two tRNAs, whose two anticodons bind in a complementary, but non‐covalent, manner to the two mRNA codons. The atomic mechanism of this self‐organization is, therefore, the base complementarity. In the two participating RNA players mRNA and tRNA, the same four RNA building blocks A,U,C,G are found in their interacting, self‐organization‐enabling parts. The interaction is named a codon (mRNA) – anticodon (tRNA) interaction. A UUU codon of the mRNA pairs with an AAA anticodon of the tRNA. In the tRNA, which in its RNA part has an AAA anticodon, the amino acid phenylalanine is covalently bound and is further activated for chemical reaction of peptide bond formation.
4. An mRNA made from six nucleotides can function as a self‐organizational unit for two tRNAs. The two amino acids as parts of the two tRNAs are thus brought into spatial proximity. This proximity effect alone brings about (catalyzes) a specific formation of a molecule from two amino acids and therefore facilitates the specific precise synthesis of a dipeptide from two amino acids. Such a scenario has only recently been achieved experimentally [75]. The bond between the RNA part and the amino acid part of the tRNA itself is chemically unstable and therefore, release of the dipeptide involves little barrier.
5. This system must, owing to the functional properties of the newly created *emergent* polypeptides as a precursor to proteins, have an advantage in selection. Within the emergence of a specific polypetide, the advantage in natural selection is transferred to the protein, so that the “RNA‐protein” world replaces the “RNA‐only” world.
6. It remains unclear and also fascinatingly puzzling from which length upwards short polypeptides perform a function and thereby possess a selection advantage. The discovery of the world of “small proteins” recently is interesting in this context (e.g. Storz et al.; Kubatova et al.) [76, 77].

**FIGURE 9:**
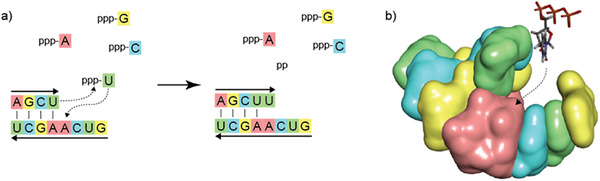
The autocatalytic, synthetic elongation of an RNA strand. (a) Two RNA strands position themselves antiparallel to each other. The choice of these two strands is not arbitrary. One finds that upwards of a length of four base pairs (here A‐U, G‐C, C‐G, U‐A), the short, double‐stranded section can assume a defined, double helix structure which is stable at room temperature (see b). There are a total of 4^4^ = 256 different RNA sequences made from four RNA building blocks. The shorter strand is referred to as an RNA primer strand. The length of the longer strand, referred to as the RNA template strand, is arbitrary. For the strand, selected here, of 8 RNA building blocks, 4^8^ = 65384 possible sequences arise. The random, simultaneous synthesis of these two partially complementary strands does not yet require the production of an astronomically large number of RNAs. The precise sequence (5’‐AGCU‐3’) of the complementary sections is not important here, only their complementarity. (b) Through spontaneous self‐organization of these two strands, a short RNA double helix is produced. It enables the autocatalytic reproduction of a complementary RNA strand. There is a chemical reason for this: the incorporation of the fifth RNA building block (in the example rUTP) into the shorter strand is sterically favored by the helical structure of the double strand (the chemical reaction mechanism speaks more precisely for stereoelectronic reasons). This steric favoring is the chemical reason for the autocatalysis. An initially randomly developing short RNA primer strand and a slightly longer RNA template strand serve as a matrix for autocatalytic self‐replication. It needs to be stated that this self‐replication often requires base catalysis; the autocatalytic self‐replication remains a very slow reaction.

In 1968, Leslie Orgel and Francis Crick jointly agreed to publish two articles “back‐to‐back” documenting the results of discussion at a scientific conference on 12/20/1966 at the British Biophysical Society in London, in which they speculated on the origin of the genetic code and/or of the genetic apparatus. The retracing of the contemplations of Crick will now be discussed. Crick initially describes the contemporary genetic code, in order, from a knowledge of that which was selected by evolution, to conclude the necessities during evolution. It should be noted here that such an approach runs the risk of succumbing to ‘survivorship bias’. However, this bias is unavoidable in *a posteriori* research. The conceptually speculative oscillation between the biochemical mechanisms found in living organisms and the properties of the participating biomacromolecules and the emergence of these mechanisms during evolution is extremely stimulating and logically illuminating and will be described in the following.

**FIGURE 10:**
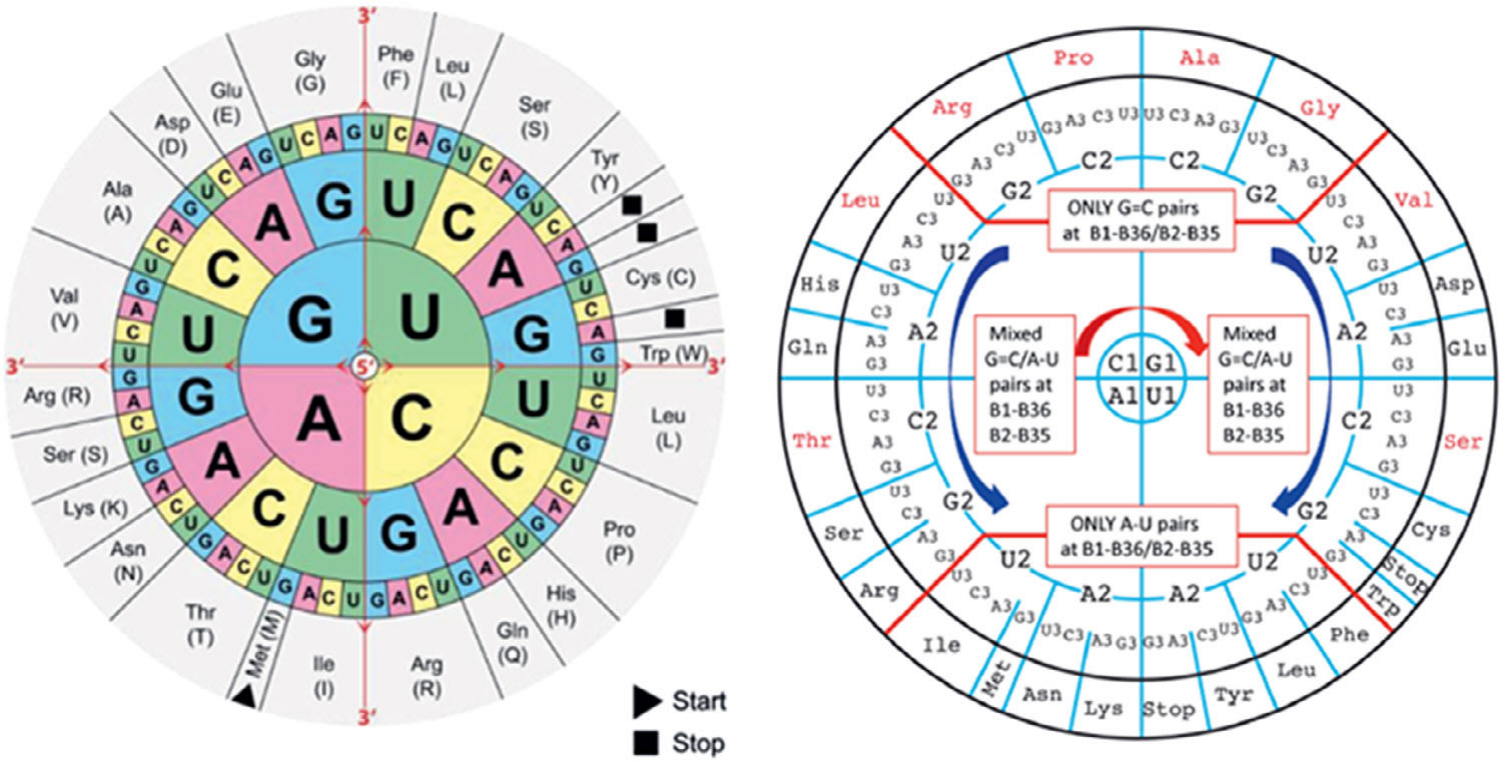
(Left): Genetic code, as depicted in textbooks. (Right): Stability‐adapted genetic code according to Grosjean and Westhof, as discussed in the text [78].

The genetic code (Figure 10) is a non‐overlapping triplet code. 61 of the 64 triplets code for an amino acid. Apart from the amino acids tryptophan (Trp) and methionine (Met), there is more than one coding triplet for an amino acid. The code is therefore degenerate, and possesses the following interesting properties:

1. The correlation between triplet and coding amino acid is not coincidental, neither in respect of the identity of the amino acid nor the fundamental physical and chemical properties such as hydrophilicity and hydrophobicity.
2. XYU and XYC triplets always code for the same amino acid (X stands for a first RNA building block, Y for a second, but different RNA building block, U and C stand for uridine and cytosine).
3. XYA and XYG triplets frequently code for the same amino acid (A and G stand for adenosine and guanosine). The triplets for tryptophan and methionine, for which there is only a single coding triplet, constitute exceptions to this rule.
4. If the two first RNA building blocks are either only G or only C, then the four triplets with the same two first RNA building blocks always code for the same amino acid. In these cases, the identity of the third RNA building block is irrelevant. This third RNA building block is then called the “wobble” codon position.
5. The distribution of amino acids on triplets is not random, e.g. all triplets with U as a second RNA building block code for hydrophobic amino acid.
6. The genetic code is universal, i.e. the same in all organisms.
  Exceptions to the universal genetic code used in all organisms are observed in non‐plant mitochondria in the cytoplasm of eukaryotic cells. This is interesting because it is consistent with the endosymbiont theory, which was mentioned by Watanabe and Yokobori, for example [79].

Crick discusses theories as to how the genetic code could have arisen. The so‐called stereochemical theory by Woese postulated a close link between the site of interaction between mRNA and tRNA, on which the information is transferred, and the site of connection of the amino acid on the tRNA. This postulated link implied that the chemical complementarity of the codon‐anticodon interaction should determine which amino acid is covalently bonded to the tRNA [80]. Interestingly, a theory was also proposed that the amino acid would have to fit the codon sequence of the mRNA stereochemically. There is no kind of experimental confirmation of this theory [81]. Whether the specificity of the codon‐anticodon interaction on the one hand and the covalent linking of subsequent amino acids is based on direct stereochemical causes has been elegantly disproved by the group of Schultz [82]. Schultz was able to develop a system of tRNA and aminoacyl‐tRNA synthetases (the class of enzyme that catalyzes the binding of an amino acid to tRNAs) which brings about the covalent bonding of a non‐natural amino acid O‐methyl‐L‐tryosine to a tRNA. This tRNA possesses the anticodon (AUC) which is complementary to one of three stop codons (UAG), the amber stop codon. If one synthesizes a gene without an amber stop codon, then the tRNA with the critical AUC of the non‐natural amino acid can be decoded by the stop codon UAG. In such *Escherichia coli* bacteria, the release factors which recognize the amber stop codon are additionally suppressed. Then the non‐natural amino acid is, in the bacterium *E. coli*, specifically integrated into proteins. These experiments show that the codon‐anticodon interaction does not have a major influence on the identity of the amino acid to be integrated.

**FIGURE 11:**
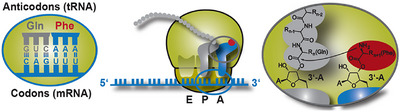
Codon‐anticodon interaction between mRNA and tRNA (left). This interaction is the prerequisite for protein biosynthesis which takes place in the ribosomes, a biomacromolecular complex of mRNA, ribosomal RNA (rRNA), tRNA and proteins (center). If the correct mRNA‐tRNA pairs find each other in what are known as the P and A sites of the ribosome (P for peptidyl and A for aminoacyl), then the spatial proximity of the two tRNAs results in the formation of a new peptide bond. Hence, the polypeptide chain, which is covalently bonded to the tRNA in the P site (symbolized by gray spheres), is transferred to the tRNA in the A site. After forging the new peptide bond, therefore, the entire nascent polypeptide chain is bound to the tRNA in the A site. In the next step of translation, the protein biosynthesis, both tRNAs are translocated in the direction of the 5’‐end of the mRNA and then occupy the E site (E for exit) and the P site. The empty tRNA in the E site leaves the ribosome and the next tRNA can bind to the A site that is free at this moment. See also Figures 18, 19, 20, 21 for the illustration of the kinetics of the individual translation steps.

At the conceptual level, the question still arises as to what argues against the evolution of a base quartet. The codon‐anticodon interaction is stabilized through the formation of hydrogen bonds (Section 4). The more RNA building blocks are part of a codon, i.e. four building blocks in a base quartet instead of three building blocks in a base triplet, the more stable this interaction becomes. During protein synthesis, at least two codon‐anticodon pairs must form in proximity to each other so that the two amino acids bound on the anticodon‐tRNAs within the ribosome can be brought into proximity and a new peptide bond can be formed (Figure 11). After this step, however, one of these two codon‐anticodon interactions must be broken again, so that the next tRNA can be loaded in the ribosome and a new amino acid can be covalently bonded to the growing polypeptide chain. Only in this way can the polypeptide chain become longer and longer. These steps of association and dissociation proceed very quickly, each tRNA typically spends between milliseconds and a few seconds in the ribosome (discussion in Section 9). The more stable the codon‐anticodon interaction, the slower the dissociation speed becomes, an evolutionary disadvantage. Evidently, the codon‐anticodon system involving a base triplet proved stable during evolution compared with a system comprising more building blocks or a system of more than four different RNA building blocks. In addition, the coding capacity does not need to be increased to be able to code for more than 20 different amino acids.

At this point, we refer to the investigation published in 2016 by Westhof: The 64 different codon‐anticodon pairs demonstrate a very large difference in their stabilities, with the UUU‐AAA pair being the most unstable (three times two hydrogen bonds, Figure 4) and the CCC‐GGG pair being the most stable (three times three hydrogen bonds) [78]. In order to balance out these differences, the RNA nucleotides in tRNAs were modified in a very specific way. The modified nucleotides differ in their chemical structure from the four RNA building blocks U, C, A and G and consequently even out the stability differences in the codon‐anticodon interaction through tertiary interactions. These considerations lead to the hypothesis that over the course of evolution initially a GC code could have developed which was then expanded through the introduction of A‐ and U‐RNA building blocks and RNA modifications [83, 84].

## 7 The Importance of Self‐Organization of Biomacromolecules in the Emergence of Life

Many of the fundamental reflections, in terms of scientific theory, regarding the self‐organization of biomacromolecules go back to Manfred Eigen [15]. His reflections begin where those considerations of Albert Eschenmoser, referred to in Section 3, stop: as the result of prebiotic chemistry compounds emerged from primitive precursor molecules, namely amino acids and RNA and DNA building blocks, which by means of repetitive polymer chemistry involving water being eliminated, can condense to the polymeric biomacromolecules: proteins, RNA and DNA. It is part of the synthesis of polymeric biomacromolecules, and it is central to cellular information transfer, that the polymeric biomacromolecules, at a given temperature, spontaneously organize themselves. This self‐organization assumes interactions between atoms or groups of atoms within and between molecules in order to permit directional formation of molecules as opposed to random interactions between these macromolecules. At a given temperature and concentration of the self‐organizing molecules, self‐organization is a spontaneous process. Nearly all biomacromolecular functions presuppose directional interactions as a driver of self‐organization and formation. The molecular prerequisites for molecules being able to assume such directional 3D structures were discussed in Section 4 for the nucleic acids DNA and RNA and in Section 5 for proteins. The process of transition from a linear chain of polynucleic acids or polypeptides to the formation of a 3D structure is termed folding. Proteins and RNAs in particular are capable of assuming a complex 3D structure, a 3D conformation. Below we shall outline the principles, in terms of scientific theory, as developed by Eigen.

“At the ‘beginning’ – whatever the precise meaning of this may be – there must have been *molecular chaos*, without any functional organization among the immense variety of chemical species” [15, 85]. The starting point for chemical and biological evolution is therefore a random accumulation of molecules. These molecules consist of different atoms that emerged after the big bang (hydrogen H, carbon C, nitrogen N, oxygen O, phosphate P) with different electronic and chemical properties. The formation of covalent and non‐covalent bonds between *different* atoms with *different* electron affinities results in molecules with an asymmetrical distribution of partial charges. Such molecules are therefore dipoles or multipoles. There exist attractive forces in compounds made up of *different* atoms with *similar* or *the same* electron affinities. These attractive forces are termed hydrophobic interactions, and they lead to strong hydrophobic molecules not dissolving in an aqueous medium. The biomacromolecules that consist of a variety of monomeric building blocks typically have hydrophilic and hydrophobic properties which build up in their different parts (Sections 4 and 5). A key component of their self‐organization is based on the balance of these two different types of interactions in folding into a 3D structure. The hydrophobic parts of a biomacromolecule will, in the 3D structure, point inwards away from the water, while the hydrophilic parts will point outwards toward the water. In an aqueous medium, molecules may attract or repel each other owing to dipole properties and hydrophobic properties. As long as these interactions between molecules are random, all possible spatial arrangements are equally unlikely. To turn the argument on its head, therefore, a prerequisite for life is an overcoming of the omnidirectional molecular, structural chaos. For this, it may have even been sufficient for the first reactions to take place on the charged surfaces of minerals.

The self‐organization of molecules enables the transfer of information. In this sense, molecules possess information in the distribution of dipoles and hydrophobic moieties based on covalent chemistry, because by this means the arrangement of atoms is fixed. In order to visualize this for better understanding, we can imagine that a molecule has three atom groupings consisting of two different atoms. One of the two atoms is partially negatively charged, the second atom partially positively charged. We indicate the position in the molecule of these different partial charges by means of an arrow, whose arrow tip points from the partially positively charged atom to the partially negatively charged atom. Owing to the primary geometric properties of the molecules, there are now different possibilities for the orientations of the arrows which correspond to a dipole, i.e. a magnet, for example: ↑↑↑, ↑↑↓, ↑↓↑, ↓↑↑, ↑↓↓, ↓↑↓, ↓↓↑, ↓↓↓. These eight possible options for the pair of atoms provides the possibility of directional arrangement of several molecules with such atom pairs: a dipole with the orientation: positive partial charge above, negative partial charge below, will prefer to associate itself with a complementarily aligned dipole. This kind of self‐organization of different molecules transfers information. At room temperature, the self‐organization is also reversible, since the forces of attraction are not large compared to thermal energy. This reversibility implies that, through self‐organization, the same macromolecular structures must always be produced, and structural formation and structural disintegration are in equilibrium.

Eigen points out that information transfer is not about the generation of information, but about how it can be coded for, transferred and decoded. In the language of molecules: What do those molecules look like which, thanks to their molecular structure, can contain information, how is information transferred through self‐organization from one molecule to the next molecule, from cells in a parent generation to cells in the daughter's generation and how is the information converted into function? Amazingly, not until the 1950s were the molecular foundations laid with the cracking of the DNA double helix structure and consequently the essential mechanism had been described in the central dogma of molecular biology [86, 87].

Key aspects of information theory are linked to probabilities. An event Z_1_ out of Z_0_ possible events of equal probability (how likely is rolling a 6 three times?) has an information content of: I = K ln Z_0_ (throwing the sequence 6,6,6 is one event out of 6^3^ = 216 possible events, K is a constant which is dependent on the number of the coding bits). Information transfer requires that this one event be correctly transferred rather than all other possible 215 events. Information transfer involves digital units; in the computer, binary units are used. A defined number of binary bits form one information unit, a byte. Information is coded in the sequence of n bytes. In order to be able to code 256 different events, one needs at least n = log_10_256/log_10_2 = 8 bits, if more than 8 bits are used to code 256 different events, then the code is degenerate.

For cellular information transfer, biomacromolecules are used. Here, the monomeric building blocks of the biomacromolecules correspond to the digital units on the computer. Through the polymerization of the monomeric building blocks into oligomers, bytes are produced which, for example, code for individual numbers (e.g. 0–9), and the polymerization of oligomers into polymers codes for any desired number in the decimal system: the larger the polymeric biomacromolecule, the greater the codable number of different events. In the information transfer originating with DNA we do not find binary bits (a binary bit has the base λ = 2) but quaternary bits (base λ = 4). These bits are the four DNA building blocks A, G, C and T. Three bits (for example the sequence GCT) are always consolidated into one byte. From these triplets, 4^3^ = 64 different bytes can be generated.

Table 2 illustrates the aspect of astronomically large numbers, as soon as one increases the number of monomeric units of a biomacromolecule. A biomacromolecule consisting of only 100 monomeric units possesses such a large number of possible sequences and hence an astronomically large capacity, for storing information. If protein synthesis were not instructed, but random, then one could never produce again a specific protein with a certain length from the multitude of possible sequences. Even if protein synthesis took place infinitely quickly, there would not be sufficient matter in the world to achieve this. Synthesis must therefore be instructed. To give a greater understanding of aspects of such instruction, we want to use the following analogy to illustrate them: randomly rolling (with a dice) any desired number sequence, a procedure which corresponds to random synthesis of what are always new biomacromolecules, has almost zero probability of leading to the same result even if one rolls enough times. On the other hand, DNA in human chromosomes consists of 3 billion DNA building blocks. These DNA building blocks can in principle code for 4^1.000.000.000^ different amino acid sequences. Even if one adds that in higher organisms the proportion of coding DNA is only ∼ 1% of the total DNA, the sheer extent for possible information has no functional significance, rather the information associated with life which is stored in molecules and their self‐organization must, in the course of its evolution through selection be linked with its (functional) history and the circumstances of its emergence.

**TABLE 2:** Number of possible different sequences N_λ,n_ of a biomacromolecule consisting of n units of a basis λ according to Eigen [15].

Examples	λ	n	N_λ_,n
Small proteins	20	100	20^100^ = 10^130^
Proteins from codons, containing only A and T	6	100	6^100^ = 10^78^
DNA chain coding for 33 amino acids	4	99	4^99^ = 10^69^
An oligopeptide of the length 12, which can randomly contain each of the 20 amino acids.	20	12	20^12^ = 4 × 10^15^

The emergence of a molecular, self‐organizing and self‐replicating system and its evolution over time are irreversible and not reproducible. The origin of life is an evolutionary process. Reference is frequently made to the fact that the evolution of life progresses, indeed must progress continuously from a historical point of view, because otherwise all more primitive forms prove to be or would have proved to be suddenly dysfunctional. Darwin's theory forms the necessary bridge between the more physical considerations of information and the evolution of species [88]. It is worth retracing this link between physics/mathematics, chemistry and biology. Central to the concept of the evolution of life is the aspect that information gains value through selection. The term selection dates back to Darwin:

“This preservation of favorable individual differences and variations, and the destruction of those which are injurious, I have called Natural Selection, or the Survival of the Fittest” [12].

Interestingly, the absence of an understanding of the molecular basis for selection was no reason for Darwin to doubt the validity of his theory: “It is no valid objection [to the concept of Natural Selection] that science as yet throws no light on the far higher problem of the essence or the origin of life” [12].

How can we explain selection on a molecular basis? Let us go back to the dice analogy, and imagine we had a tetrahedral dice on each of whose four surfaces there is one of each of the four DNA building blocks A,T,G,C. Imagine we were to have a game of dice. Let's assume a game master knows the sequence, but the players do not. The first rule of the game would be that we roll the dice until we find precisely one of the 4^30^ possible sequences of a DNA macromolecule of n = 30 building blocks. This boring rule results in a very tedious game and shows that no single biomacromolecule of a certain sequence and function can have accumulated through chance, through rolling of the dice, out of all other possible sequences. Through a simple modification of the rules of the game, the game becomes faster: whenever we have correctly rolled the position of one building block, we retain, thanks to the announcement of the game master, this result. We therefore introduce a selective advantage, derived from the *a‐priori* knowledge of the correct sequence. Because for DNAs, n/4 of the positions are rolled correctly by chance, the number of necessary rolls decreases on average to 4(n‐n/4) = 96 rolls. In our imaginary game, we have applied as a selection criterion that the correct sequence is known in advance. In natural systems, it goes without saying that the selection is complex, in particular, an individual sequence position and its impact on the selected property is always dependent on other building blocks in a complex manner.

Within the evolution of life, three different phases now play a key role:

1. The prebiotic phase.
2. The phase of self‐organization of prebiotic molecules, in order to create self‐replicating systems.
3. The evolution of individual organisms.

The evolution of individual organisms shall not be discussed here. This third phase encompasses breathtakingly fascinating questions of cell differentiation, the development of sexual reproduction, the emergence of the nervous system, the field of autonomous decision and communication for example as a prerequisite for awareness of self and logical reflection. These aspects are researched in the disciplines of biology, medicine, psychology, sociology etc.

Below, we want to discuss the evolutionary theory of matter proposed by Eigen, in other words the transition from “non‐life”, e.g. minerals, to “life”, e.g. bacteria, plants, animals. Stegmüller points out that Eigen does not eliminate “the scope for vagueness in the terms inanimate and animate […] in order to be able to word the question with the aid of more precise terms. Rather, in both cases, constructs are used which lie beyond the scope for vagueness, and the question is posed as to how the transition from constructs of one type to constructs of a different type can be explained” [21]. For this transition to self‐replicating systems, the self‐organization of prebiotic molecules is essential. The system in which evolution takes place contains the prebiotic molecules activated for chemical reactions, in particular amino acids and nucleic acids, and also a constant energy input in the form of heat and light (both from the sun). This energy input fundamentally constitutes the driving force for shifting this system into an open state of non‐equilibrium.

At this point, we wish to briefly comment on energy as constant input for the emergence and evolution of complex systems. Ilya Prigogine, awarded with the Nobel Prize in Chemistry in 1997, linked complex systems to dissipative structures [89]. He discussed that living (complex) systems are non‐equilibrium systems “for which irreversible processes may lead to a new type of dynamic states of matter which I have called ‘dissipative structures’.” Dissipative structures are self‐organizing and complex. They form in open systems away from thermodynamic equilibrium. Through exchange of energy and matter with the surroundings, local order can be generated by taking up (solar) energy input. Irreversible processes can generate entropy and lead to what Prigogine calls “one‐sidedness of time”. The positive time direction is associated with the increase in energy.

In the subsequent discussion, we shall, as of now, replace DNA with RNA, because the effects described here for RNAs have been proven experimentally. In such a system, longer strands of RNA can be synthesized. With regard to the generation of self‐replicating systems, specific synthesis steps must be favored by means of autocatalysis. This happens *spontaneously* as follows: an initially formed strand of eight building blocks of the sequence 5’‐AACCGGUU‐3′ accelerates the synthesis of an identical second strand (Figure 9). Assuming that this specific RNA strand is capable of such autocatalytic self‐replication, it leads to an increase in the concentration of this specific RNA strand from the large number (4^8^ = 65536) of possible RNA strands. For such preferred autocatalysis to happen, the self‐replicating RNA strand must have preferred properties in order to “survive” over the other possible RNA strands from eight building blocks, i.e. it must be selected as the “fittest sequence”. Selection must be based on a functional property inherent to this chemical molecule. Such functional property could include, for example, a higher stability at a given temperature compared to the other sequences. The molecular foundations for such self‐replication and also for the inheritance of genetic features have their exclusive origins in the chemical structure of RNA. The evolution of matter can be fully explained by means of physical and chemical laws. Eigen's theory thereby provides the fundamental basis for Darwin's theory, the gap admitted by Darwin himself. This theory is also quantitative in the same sense as classical mechanics, thermodynamics and quantum mechanics.

To illustrate the subsequent course of evolution of a self‐replicating system, Eigen introduces a box that provides a barrier between an internal system and the environment. The molecules within the box are in dynamic equilibrium with their environment. In this box are monomeric building blocks, e.g. the four RNA building blocks in their activated form capable of polymerization, i.e. RNA‐**t**ri**p**hosphates (rNTPs, with N being used as a letter for each of the four nucleobases). In addition, the system has a constant supply of energy, e.g. particularly in the form of very reactive rNTPs. Each spontaneously generated strand has the capacity for self‐replication. The sequence of the building blocks in each of these polymers contains information, in other words the polymers are carriers of information. The synthesis of the polymers and their disintegration are at equilibrium. In this situation, the presence of polymers remains constant, but not the information present in its sequence. *Without interactions* (and ignoring – which is essentially justifiable – different chemical reactivities and stabilities of the *individual* reactive rNTPs), each sequence is formed with the same probability, each type of information disintegrates in this box. By contrast to this, *through self‐organization based on interaction between particles*, and through mutations, a few sequences will assert themselves thanks to their more stable properties. The autocatalysis of initially form oligomeric strands increases rapidly in the transition from dimer, trimer, and tetramer to an RNA duplex strand made from RNA primer and RNA template (discussion above). This selection on the basis of self‐organization is an emergent process, which inevitably takes place, but is not predictable: it cannot be predicted which RNA duplex strands initially emerge randomly, which *random* mutations occur owing to imperfect synthesis, nor can it be predicted which polymers are ultimately selected. The unpredictability of the selected polymer is fundamentally caused by the fact that the number of possible polymers upwards of a given number of monomeric building blocks in the polymer is astronomically large (Table 2).

At this point in the discussion, Stegmüller draws attention to the fact that it is actually only Eigen's evolutionary theory of matter which first gives quantifiable meaning to Darwin's concept “survival of the fittest”, by quantifying the term “fittest” by defining a value function. Darwin's principle was criticized as being tautological, that he said nothing other than the “survival of the survivor”. It is possible to talk of the “fittest” without moral qualms when referring to the evolution of molecules with functions, but without the properties of organisms. Poser remarks on this: “Despite the randomness and despite the independence of the elements of the triad [mutation/selection/retention], these are viewed as part of a classification system. However, by no means is every change considered a mutation, rather, such a variant which refers to a possible selection. Conversely, a selection is not some kind of positive/negative separation but rather one that is caused by a mutation. So, with the triad, as emphasized by Niklas Luhmann, it is a case epistemologically speaking of ‘corresponding terms, which are not used outside of evolutionary theory.’ In this respect, the independence of mutation and selection experience a system‐ or model‐specific limitation, which in each case must be identified. In particular, it would be naïve to see in mutations a description of that which exists, for example, of Nature. Viewed epistemologically, this is a classification structure first imposed from outside” [22]. Poser's introduction of Nature as something that exists and could possess properties outside of that which can be described using natural science is, however, astonishing from a natural science perspective.

For a deeper understanding of the evolution of matter in the box described above, the following aspects are important: In the box, a large number of n polymers with different sequences is formed (P_1_, P_2_, …, P_n_). The change in concentration over time, dx*_i_*/dt, of a polymer P_i_ is determined by different factors (Equation 1).

(1) $$\frac{dx_{i}}{dt}=\left(F_{i}-R_{i}\right)\times x_{i}+\sum_{i\neq k}Q_{\mathrm{ik}}\times X_{k}$$

The constant F*_i_* describes the self‐replication of a polymer P*_i_*, and the constant R*_i_* the disintegration of the same polymer P*_i_*. The self‐replication F*_i_* is dependent on the two factors A_i_ and Q_i_, and the disintegration constant R*_i_* on the two factors D*_i_* and V*_i_*:

(2) $$F_{i}=A_{i}\times Q_{i}$$

(3) $$R_{i}=D_{i}\times V_{i}$$

A*_i_* is the reproduction rate and Q*_i_* is a quality factor for the reproduction. For Q = 1, the i‐th polymer is reproduced with no errors. The smaller Q*_i_* is, the greater the error of reproduction. Each polymer possesses a different reproduction rate and a different quality factor. Reproduction errors lead to a mutant k of the starting sequence, i.e. a polymer P*_k_* (k≠i) of a different sequence. The total number of mutants which can form within a time interval from a starting sequence is given by the product A*_i_* *(1‐Q*_i_*). The distance of two sequences of polymers P*_i_* and P*_k_*, which converts P*_i_* via mutation into P*_k_*, is referred to as the Shannon distance. Two polymers with 3 mutations have a Shannon distance of 3. The Shannon distance is very small compared to the astronomically large number of possible sequences. An RNA polymer made up of 100 RNA building blocks has 4^100^ = 1.6*10^60^ different sequences; the number of necessary mutations, i.e. the maximum Shannon distance between two sequences is 100 = 10^2^. In other words, it requires a maximum of 100 exchanges to convert every possible sequence into all other possible sequences. In this system, therefore, a mutation is initially the integration of an incorrect monomer. In the system described here, mutations occur spontaneously and are not generated through exogenous factors such as nuclear radiation or the influence of mutagenic substances.

In Equation (3), D*_i_* gives the disintegration rate of the i‐th polymer and V*_i_* a possible dilution of these sequences. Ultimately, the sum in Equation (1) takes into consideration that via the mutation of a polymer P*_k_* specifically P*_i_* can be generated once again. The probability of this occurring depends on the Shannon distance between the polymers P*_k_* and P*_i_* and the quality factor Q*_k_*.

By way of a reaction environment in which such an imaginary experiment can be implemented, the notional box could be in flow equilibrium with the external world; in which e.g. smaller polymers with fewer building blocks, and therefore a smaller size, could diffuse out through size‐selective pores. In an experimental set‐up, which models such a situation, a box with semi‐permeable walls is located in a large bucket in which the activated monomeric RNA building blocks are present. The following reactions would take place in the box:

(4) $$\mathrm{ATP}+\mathrm{GTP}+\mathrm{UTP}+\mathrm{CTP}\to \mathrm{different}\;\mathrm{RNA}\;\mathrm{strands}+P_{2}{O_{7}}^{4-}$$

The starting compounds of triphosphates arose in the course of prebiotic chemistry. The RNA strands now reproduce by means of autocatalysis (Figure 9): randomly, an RNA strand of the sequence 5'‐GCUAUAGC‐3' and a second shorter strand 5’‐GCUA‐3’ could have been generated. The second strand is complementary to the 5' end of the longer strand and would, through self‐organization, form a dimer of two strands, which consists of the two different molecules (Equation 5). In this dimer, the subsequent polymerization reaction from the 4mer to the 8mer RNA strand is favored through self‐organization; it becomes quicker via autocatalysis (discussion in the figure caption of Figure 9) [90]. When randomly selecting the sequences of the two strands, we have selected the sequences in such a way that the newly emerging strand, the daughter strand, is identical to the parent strand, since we started with a self‐complementary, palindromic sequence of the 8mer parent strand of RNA. Through the autocatalytic, self‐replication of the RNA, the number of identical RNA strands doubles, whereas in the case of non‐palindromic RNA strands this occurs after not one but two synthesis cycles:

(5)

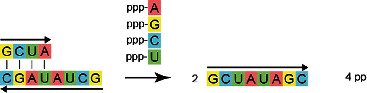




The capacity of RNA for self‐replication is based on the possibility of self‐organization, which for RNA and DNA is based on complementarity in the number of hydrogen bond donors and acceptors of A and U or C and G. The efficiency of the self‐replication is based on the autocatalysis step, which is preceded by the addition of two complementary strands (4mer and 8mer RNA). The addition of further monomeric building blocks to a short double helix is many times more efficient than the extension of just one strand of RNA at the 3’‐end [90]. It should once again be emphasized, however, that the capacity for self‐replication and for autocatalysis is based on the inherent chemical properties of the RNA building blocks. They are, accordingly, a physical and chemical necessity. Also, faulty self‐replication, which leads to a mutation, is not purely coincidental but depends on physical and chemical properties such as the relative concentration of the monomeric RNA triphosphates, the rNTPs, and the affinity of a new building block for the existing single strand and/or double strand of primary RNA and template RNA. These properties permit quantifiable predictions of the error rate. When a mutant occurs, however, it is not predictable, but stochastic. Even the simple reaction systems possess elements characteristic of living systems:

4. Anabolism and catabolism, i.e. the construction and degradation of substances inherent to the system, are brought about through the uptake of the energy‐rich RNA triphosphates and the separation of diphosphates (Equations 4 and 5). Because of the semi‐permeable membrane, the box is an open system, owing to the non‐feasibility of all possible RNA sequences and the autocatalytic properties of self‐replication of the initially emerging RNA strands, the system is irreversible, since the polymerization is accelerated through the autocatalytic effect in different ways [91]. The solar energy provides the energy input for the synthesis of activated RNA triphosphates, in which the thermal solar energy is converted into chemical energy and stored.
5. The complementarity of the RNA building blocks enables self‐replication, individual RNA strands can unequivocally pass on their information or also identity.
6. Faulty self‐replication is the molecular basis for mutations, which introduces variability into the replicating strands. The ratio of mutation to precise replication becomes essential for both evolutionary adaptability and the survival of identity, as described later.

In these primitive living systems, segregation and selection can take place. Segregation describes the ratio A*_i_* *Q*_i_* to D*_i_*. Polymers with A*_i_* *Q*_i_* > D*_i_* survive, whilst polymers with A*_i_* *Q*_i_* < D*_i_* die out. Over and above segregation, selection pressure may be generated which can arise through the changing external parameters, the general concentration of all RNA triphosphates or through restriction of supply of individual RNA triphosphates. At this point, the concept of excess production E*_i_* = A*_i_* – D*_i_* of the polymer P*_i_* is introduced, relative to the average production E**_0_**, the mean value for concentration of all polymers. The polymers are assigned a selection value W*_i_* = A*_i_* *Q*_i_*‐D*_i_*. The polymers fall into two categories: only polymers with E*_i_* > E_0_ reproduce. The selection value therefore defines a threshold value, which reflects the ratio of construction to disintegration of a given polymer. In a system of stationary supply of starting compounds, the autocatalytic property of self‐replication means that the polymer diversity in the system breaks down until, in a threshold case of exact reproduction, only one single polymer with the largest selection value remains. As time progresses, the polymers evolve into an ensemble in which, as the case may be, scarce numbers of polymers mutated at certain points coexist. Such an ensemble could for example consist of 90% identical polymers with what at this point in evolution, under these current environmental conditions, are optimum sequences, 5% different polymers of in each case only one, but different mutation, 3% polymers with two mutations and 2% additional polymers with a higher number of mutations. If the number of polymers in the ensemble is large, then at least one copy of each single mutant can be present. For such an ensemble, Eigen introduced the term quasi‐species; its experimental confirmation is discussed below. Such a quasi‐species has the evolutionary advantage of being able to react much faster than an ensemble consisting of 100% identical polymers to a changing selection pressure. The selection value constitutes a relative optimum, which has evolved where selection exists. The fact that an absolute optimum can effectively never be achieved is due to the incomplete coverage of the almost astronomically large sequence space. It thereby represents a relative optimum within the space of the biologically possible worlds. The analogy of the expressions “biologically possible worlds” refers back to Leibniz's “best of all possible worlds” and confirms the marked way in which complexity research is based on Leibniz's philosophical principles [92, 93].

In Eigen's evolutionary theory of matter, there is therefore a precise, quantifiable definition of the “survival of the fittest” which can be assigned to that polymer which “whose selection value, versus its rival” is superior when selection pressure exists [21]. There are limits to the optimization of the selection value in this simple, self‐replicating system. Each chemical reaction is reversible. In addition, at each polymerization step, e.g. through the incorrect integration of UTP instead of CTP during the chemical reaction outlined in Equation 5, a side reaction may occur. The correct replication of the polymer P*_i_* becomes more difficult the longer the polymer is. The quality factor Q*_i_* of the polymer P*_i_* of a length n depends upon the correct integration in each lengthening step q: Q*_i_* = q^n^. At a length of 100 and an assumed correct integration q of 0.99 (99% correct integration), the result is 0.99^100^ = 0.37, in other words only 37% of the polymers generated are actually the correct polymer P*_i_*. The information content of the self‐replicating system can, however, only be achieved by increasing the length of the polymer. In contemporary living organisms, the reproductive accuracy during replication is 99.9999%; this is achieved through precise integration and through a subsequent error check; with transcription and translation, the values named after quality control are 99.9–99.99%. Replication, i.e. the passing of the DNA to the daughter cell, must be much more precise than transcription and translation. This accuracy, however, could not be achieved during evolution by RNA molecules themselves. Hence, the emergence of proteins as efficient catalysts of replication is the key prerequisite for lowering the error rates, and therefore for producing more complex life in the sense of life that contains more information.

Eigen's model of evolution of matter also provides the framework for this, for the expansion of an “RNA‐only” world into a “Protein‐RNA” world, with the introduction of coupled hypercycles. The chemical possibility of linking RNA and amino acid building blocks, and the emergence of the genetic code, were the prerequisites for this evolutionary step toward higher organisms. In the interests of continuous evolution, the genetic code, i.e. the correlation between coding base triplet and coded amino acid, developed randomly. It can be assumed that initially not all possible amino acids of prebiotic chemistry were coded for and the existence of a triplet code first emerged from GC‐RNA building blocks, as already indicated.

The development of a coupled hyper‐cycle is breathtakingly exciting, “because here, extraordinarily, *logical* necessities, chemical‐physical *laws* and ‘*mechanistic coincidences’* had to interact” [21]. In this emergent step, the syntheses of two polymer categories capable of polymerization, RNA and proteins, are coupled to one another. Random mutations at the level of the RNA result, through the synthesis coupling, in proteins with different sequences and hence a different 3D structure and properties. Only by these means can selection, which acts on the properties of proteins, become effective through the mutation of RNA building blocks. The reason for the logical necessity is that a base triplet can code for 20 different amino acids. The code is based on the self‐complementarity of the RNA building blocks, the reversible formation and the dissociation of base pairs; these are geometric necessities for being able to form regular two‐ and 3D structures. Mechanistic coincidences relate to the initially random links of amino acids to base triplets, which have evolved into a stable system.

A hypercycle of RNA sequences and covalently bonded amino acids, which can form proteins, consists of polymeric RNA information carriers, which code for protein chains. The RNA information carrier is reproduced by means of self‐replication based on self‐organization. The self‐replication of one of the information carriers can then be selectively improved if protein chains have emerged which accelerate the self‐replication and, as appropriate, also improve their accuracy. Through these improvements, longer RNA information carrier molecules can be synthesized which, in turn, can code for longer protein chains. A system in which such a hypercycle of a “RNA‐protein” world can develop will displace an “RNA‐only” world. The hypercycle systems also obtain a selection advantage when they separate themselves off from the world around them by means of an envelope, as was implemented in the organisms through enclosure with a membrane envelope. There are differences of opinion over the chronological sequence of the individual steps. In Eigen's model, the development of a membrane envelope is conceptually simple, whilst the evolution of a hypercycle is a mental challenge. For this reason, Eigen concerns himself less with the development of a cell membrane, which permits a separation between inside and outside, i.e. a cell membrane that seals off the world. Eigen's theory is quantitative and also compatible with the thermodynamics of open, irreversible systems, an aspect which cannot be discussed further here.

We want to conclude this section with the groundbreaking experiment by Spiegelman, which investigated the evolution of the Q_β_ bacteriophage under selection pressure in vitro [94, 95]. The significance of the development of this system is that Eigen's evolutionary theory, which provides precise predictions on the basis of its quantitative character, can be checked experimentally. By this means, one avoids the criticism that studying evolution is inevitably historical and thereby can be reduced to not having to be studied, since it is obvious that life in its current form has emerged. From the Q_β_ bacteriophage, Spiegelmann isolated an RNA polymerase, which catalyzes the replication of the Q_β_ bacteriophage RNA. Thus, he isolated both of necessary molecules that characterize a hypercycle, in order to investigate in vitro how the RNA of the Q_β_ bacteriophage evolves under selection pressure. The design of such an experiment shall be briefly described here. Initially, 250 µL reaction solution receives 0.2 mg of the bacteriophage RNA, 40 µg of the bacteriophage RNA polymerase and the activated RNA building blocks. The RNA is reproduced by means of catalysis by the RNA polymerase for 20 minutes at 35°C. Then 20 µL are withdrawn and transferred into fresh reaction medium, which contains all components apart from the RNA. The source of the RNA therefore comes exclusively from the first batch. Since only 8% of the volume of the first reaction batch is transferred, a specific RNA must have been replicated around twelve times within the 20 min of reaction time to be inserted. The more frequently an RNA has been able to replicate, the greater the chance of this RNA of surviving the transfer. Through this experiment, by way of selection pressure, the replication rate is increased: Spiegelman was able to show that within 14 hours, in which 74 transfer steps were carried out, the length of the surviving RNA genome was reduced to 17% of the initial genome length. In addition, the integration rate per RNA building block is increased by a factor of 2.6. Both bring about a 15‐fold acceleration of the replication rate of the shortened RNA genome. Modern experiments make it possible to follow the progress of the mutations in every cycle to draw conclusions about which mutation has brought about the selection advantage.

Experiments performed later by Weissmann also use Q_β_ bacteriophages and form an experimental basis for the concept of the quasi‐species introduced above [96]. In these experiments, the solution is dissolved in the starting reaction vessel to such an extent that in the subsequent reaction mixture there is, on average, always precisely one single phage particle per reaction vessel. By this means, all the following steps and each subsequent dilution step become monoclonal with respect to the bacteriophages. The result of the genome analysis of the replicating bacteriophages is that the error rate of the replication is 3*10^−4^. The RNA is therefore heterogeneous and on average exhibits 1–2 mutations. Weissmann writes: “We propose that the Q_β_ phage population is in dynamic equilibrium, with viable mutants arising at a high rate […] on the one hand, and being strongly selected against on the other. The genome of Q_β_ phage cannot be described as a defined unique structure, but rather as a weighted average of many different individual sequences.” Thus, Weissmann had established the experimental basis for the concept of the quasi‐species, a term coined by Eigen.

## 8 Folding of Biomacromolecules: From Information to Function, Using the Example of Protein Folding

In Section 7, we saw that the existence of an “RNA‐only” world was replaced by the coexistence of RNA and proteins, whose coevolution was coupled through the existence of hypercycles. In a later step, the protein ribonucleotide reductase evolved, which converts RNA into DNA, creating the prerequisite for the creation of an RNA‐DNA‐protein world. The evolutionary advantage of an RNA‐DNA‐protein world lies first in the greater stability of DNA as a permanent store for genetic information, and second in the higher functional variability of the proteins compared with RNA. Except for intrinsically disordered proteins, a concept only introduced relatively recently, the function of a protein is dependent on its 3D structure, its conformation [97]. The acquisition of this 3D conformation is referred to as folding. The necessity of folding to adopt a conformation in which a protein can exert its function thus constitutes a key extension of the central dogma of molecular biology and will now be discussed.

Which conformation is adopted by a given sequence of amino acids? Experiments by Anfinsen document that a given sequence of amino acids always adopts the same 3D conformation. This conformation is referred to as the folded state or “native state” of the protein. For monomeric proteins, this folded state is the most thermodynamically stable state which can be adopted by the polypeptide chain of a given amino acid sequence, i.e. that with the lowest total energy composed of different molecular interactions and entropic contributions [97].

The folded state can be described geometrically by the position of all individual atoms. Here, two atoms cannot possess the same coordinates [98]. Each atom has a certain extension (van‐der‐Waals radius). Through the mutual repelling of the electron shells, the atoms cannot come too close to each other. If we assume that an amino acid, on average, consists of 10 atoms and a folded protein is constructed from 100 amino acids, then the folded state is described by 10*100 = 1000 x,y,z‐coordinates. Each atom *i* has, at equilibrium, one coordinate K, one atom position: atom_i_ → K(x_j_,y_j_,z_j_). This coordinate is subject to fluctuations owing to the thermal energy. In the folded state, the fluctuations have a low amplitude. The possible conformations are subdivided into macro‐states and micro‐states. A ‘macro‐state’ is defined as a state that is separated from another macro‐state by an energy barrier. For example, one might consider the “native state” and the “almost native state” to be two such macro‐states. The height of the barrier governing their interconversion depends on temperature. A macro‐state can also break down into microstates, which include various non‐folded configurations, for example. Occupying different micro‐states with elements of a system is preferable in entropy terms, while two different micro‐states are not separated by an energy barrier [99].

There are two aspects that make protein folding a problem of complexity, for long, both problems have been considered as paradigm for complexity on the level of molecules.

*Thermodynamic problem of protein folding*: Based on the experiments by Anfinsen, one should be able to predict the coordinates of the atoms in the folded state (“native state”) as the most thermodynamically stable conformation for any desired protein sequence. However, folding requires the interaction of a vast number of interatomic interactions between the atoms of the protein and their interaction with the solvent water, which for long remained close to impossible to solve. To describe aspects of this puzzle, simpler model, in particular the HP lattice model discussed below, have been developed. This HP lattice is very illustrative to explain the nature of the problem in a transdisciplinary discourse.

In the last few years, breath‐taking progress has been made in this field, particularly through methods of *machine learning*, with the program AlphaFold being award the Noble prize for Chemistry in 2024 [100]. Machine learning involves approaches in which a program is trained, by means of experimentally determined protein structures and homologous sequence relationships from related organisms (two evolutionary conserved co‐mutations indicate interaction of the two mutated amino acids in the protein structure), to recognize patterns and apply these to unknown protein sequences. Unlike earlier methods, *machine learning* approaches are also impressively accurate for those proteins for which no structural data for closely related proteins exists (*De‐novo* structural prediction).

On a fundamental level, such approaches do, however, not provide any description of the folding process, and the size of the training data sets is, compared with the number of all natural protein sequences, or indeed all conceivable protein sequences, infinitesimally small. Consequently, the newest approaches are also probably limited in their applicability to a subset of protein sequences which, in some fundamental properties, are similar to the structures previously solvable experimentally. According to Anfinsen, however, the sequence alone should be sufficient to predict the folded state. Prediction of the folding process (including the kinetics of folding and prediction of folding intermediates) has so far not succeeded because the interactions between the amino acid side chains within the protein (Figure 6) and the interaction of these side chains with the solvent or their enthalpic (ΔH) and entropic contributions (ΔS) cannot be fully described. The problem presents itself as follows: For 129 amino acid side chains of a protein there are (129*128)/2‐pairwise interactions which occur between one or more atoms of this amino acid side chain. These interactions are, in different ways, dependent on the distance and the orientation of the atoms. In addition, the strength of the interactions is dependent on the solution environment. There are, in particular, (attractive and repulsive) charge interactions between the polar amino acids and van‐der‐Waals interactions between the hydrophobic amino acids (Figure 6). Charge interactions are described by Coulomb's law, the interaction of two charged amino acids is dependent on the dielectric ε_0_ of the solvent. Consequently, the interaction of two charged amino acids depends on whether their side chains are located on the surface of a protein or in the interior, inaccessible to solvent. Hydrophobic interactions show distance‐ and orientation dependencies which are more difficult to quantify. Unlike RNA and DNA polymers, in which the interactions between individual building blocks are easy to describe, the interactions in proteins are, so far, not even capable of being fully described using quantum mechanics methods.

**FIGURE 12:**
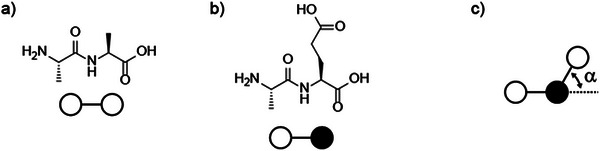
Reduction of the representation of an amino acid within the context of the HP lattice model. An amino acid is represented by a bead. The properties of the 20 different amino acids are divided into only two different categories: polar (P, unfilled circle) and hydrophobic (H, filled circle). The peptide bond is represented by a line. The dipeptide L‐Ala‐L‐Ala‐OH depicted in (a) has two amino acids with two hydrophobic side chains, and the dipeptide L‐Ala‐L‐Glu‐OH depicted in (b) has amino acids with one hydrophobic and one hydrophilic side chain. The angle α indicated in (c) shows the relative orientation of the amino acids.

*HP (hydrophobic‐polar) lattice model for a mathematically precise description of the protein folding problem*: In order, therefore, to make the quantifiable aspects of the protein folding problem easier to understand, a descriptive model shall be discussed here (Figure 12). This model drastically simplifies the folding problem. It has the advantage that within this simplified model, each step, each possible conformation, can be described precisely. The model is based on the following considerations and simplifications: proteins are linear, polymeric molecules constructed from amino acids, its 3D conformation, is stabilized by non‐local interactions, i.e. interactions between two amino acids which are not directly adjacent. The simplified HP lattice model discussed here is very descriptive in the context of an interdisciplinary discussion of complexity. The model assumes that one amino acid is represented by a circle (2D model) or a sphere (3D model) and that the sequential distance of the covalent bonds between two amino acids is illustrated by a joining line (beads‐on‐a‐string‐model; Figure 12) [101].

**FIGURE 13:**
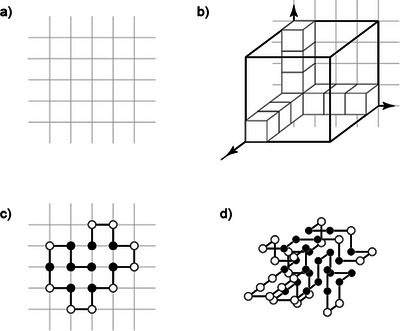
(a) Lattice points of a 2D lattice. (b) Construction of a 3D lattice. Images from the Internet. (c) A conformation of a polypeptide chain in the 2D HP lattice model, (d) a conformation of a polypeptide chain in the 3D HP lattice model, modified version of Lau and Dill [101].

**FIGURE 14:**
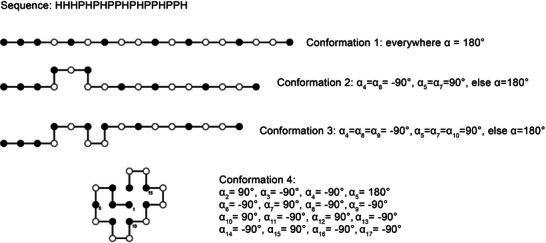
Four of the possible conformations of the polypeptide chain with the sequence HHHPHPHPPHPHPPHPPH. Conformation 1 represents that particular conformation which has the greatest “end‐to‐end” distance (18*0.38 = 6.84 nanometers). In this conformation, all angles are α = 0°. For Conformation 2, the following applies: α _4_ = α_8_ = −90°, α_5_ = α_7_ = +90°. The number of attractive H‐H interactions is the same in Conformation 1 and Conformation 2, because both conformations only display interactions between adjacent side chains that occur in the same way in all conformations. Conformation 3 displays three branching angles: α_4_ = −90°, α_7_ = +90°, α_9_ = −90°. By this means, both side chains 7 and 10 are on adjacent points of the 2D lattice. Conformation 3 is hence stabilized vis‐à‐vis Conformation 1 and Conformation 2 by (‐) one energy unit. Conformation 4 of the polypeptide chain is determined by the values of 16 angles α. The chain is very compact. It has nine attractive H‐H interactions (2–5, 3–18, 7–2, 7–10, 10–1, 12–1, 12–15, 15–18, 18–1). The number of possible conformations that have nine attractive H‐H interactions is much smaller than the number of possible conformations which display one or two attractive interactions. Conformation 4 is stabilized thanks to these nine interactions (enthalpic stabilization), Conformation 3 and similar conformations are stabilized with just one interaction owing to the number of possible conformations (entropic stabilization).

The different amino acids are assigned only two different properties instead of twenty different ones (Figure 6), either an amino acid is hydrophobic (H) or polar (P), in the Figures 12, 13, 14 this is symbolized by empty circles and circles colored in black, respectively. The relative position of three spheres in the 2D space is then described by an angle α.

The spheres can now be positioned on points on a lattice that are separated from one another by uniform lengths. Here, lattice points can be occupied or unoccupied by spheres. There are 2D and 3D lattices. In the 2D lattice (Figure 13a), the spheres are in one plane and the joining line between two spheres can be moved either upwards/downwards or to the right/left (the angle α can only assume the values 0°/−180° or ±90°). In the 3D lattice (Figure 13b), the spheres are located in a cuboid, and joining lines may additionally be extended forwards or to the rear (the position of the adjacent spheres is thereby determined by two orthogonal angles). All conformations that can be represented in a 2D lattice are also present in a 3D lattice. In the 2D lattice there are z = 4 adjacent points for every point on the lattice, the number of possible orientations of the bonds is z‐1 = 3. For the 3D cubic lattice, it follows that: z = 6. The atoms C_αi_,C’_i_,N_i+1_, C_αi+1_ are in one plane owing to the planarity of the peptide bond. Their distance from each other is constant. For this reason, they are represented in the HP model by a virtual line joining the two C_α_ atoms. The distance between the two C_α,_ *_i_* and C_α,_ *_i+1_* atom, one refers here also to the virtual C_α,_ *_i_* and C_α,_ *_i+1_* atom, is 0.38 nanometers. The representation of this virtual bond through a line of uniform distance in the HP model is therefore not a gross simplification. However, the HP model is a gross simplification with respect to two stipulations. First, the relative orientation of two side chains is significantly restricted. In the 2D‐HP lattice model, this is reduced to an angle α, for which the values 0° or ±90° are permitted. However, one can imagine simple extensions, how one can progress from square box lattices to more interesting lattices. Second, the possible interactions between two amino acids are heavily restricted. This constitutes the biggest simplification in the HP models. Each of the twenty amino acids is built from different atoms, so that the interaction fields originating from each amino acid are different. These are compensated for by the 3D arrangement of the amino acids in space, e.g. by side chains with opposing charges attracting each other and side chains with the same charge being as far away from each other as possible. By contrast, there are only two types of amino acids in the HP model, namely hydrophobic (H) or polar (P). Interaction terms are assigned to the interaction of these two possible properties of hydrophobic or polar. In the simple HP lattice model by Dill et al., the relative energies (HH, PP, HP = PH) = (−1,0,0) apply, but other allocations have also been proposed [52, 101]. A negative energy term is stabilizing. One sees that in the HP model according to Dill, the stability of a protein is brought about exclusively by the number of contacts/interactions between two hydrophobic amino acids. The sequence of a polypeptide chain is defined by the sequence of hydrophobic or hydrophilic amino acids. The chain shown in Figure 14 has the sequence: HHHPHPHPPHPH PPHPPH. For such chains, all conformations can be generated. Figure 14 shows possible conformations of the polypeptide chain. Regarding terminology, earlier literature sometimes uses the term configurations, which may lead to confusion. In accordance with the IUPAC Committee on Chemical Terminology, the term “conformations” is preferred. They vary with regard to the individual angles α. As is clear from the diagram, Conformation 3 is more compact than Conformation 2, which is more compact than Conformation 1. The “end‐to‐end” difference is different in the three conformations. In addition, one notices on this construction that the number – according to the model – of solely attractive interactions (H‐H‐interactions) is, for interactions between two adjacent side chains, the same in all conformations and therefore does not need to be counted. The interaction occurring in Conformation 3 between the side chains 7 and 10, which are precisely one lattice point away from each other, is the first stabilizing interaction. On this basis, all the possible conformations can be designed. After designing (including on the computer), their stabilities can be calculated thermodynamically, i.e. with respect to their enthalpy and their entropy. The model generates e.g. the energy of the different conformations from the number of HH contacts; the number of accessible conformations is produced from an increasing compactness, induced by the increase and compression of the HH contacts.

**FIGURE 15:**
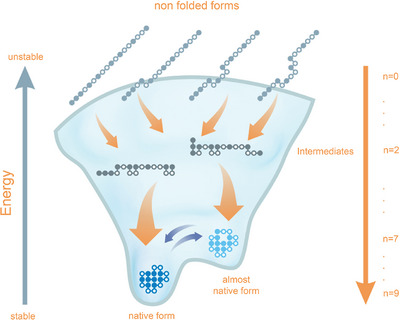
(top) Application of energy as a function of native contacts. In the lattice model, by way of simplification, only hydrophobic contacts are assumed. On the left‐hand energy axis, the energy decreases as the more contacts n (right) are formed. The number of possible structures decreases the more native contacts are present. Structures with fewer contacts are favored, entropically speaking.

Figure 15 illustrates the process of protein folding. The energy landscape of a folding protein is depicted by a funnel. Under conditions (of temperature and chemical environment) in which a protein can fold, the folding progresses based on elongated conformations without HH contacts (n = 0) until reaching the most thermodynamically stable conformation with maximum HH contacts. On the upper edge are several possible forms of the polypeptide chain without HH contacts. Initially, what are still relatively unstable folding intermediates form, with few (n = 2) HH contacts. There are still many different shapes with two HH contacts. During the folding, the folding funnel narrows; this is an indicator of the number of different shapes (conformations) of the polypeptide chain. Starting with intermediates with two HH contacts, two different, very stable forms can be generated: an almost native form with seven HH contacts, and the most stable, native form with nine HH contacts. As can be derived graphically from Figure 15, the arrangement of the seven HH contacts in the almost native form is significantly different from the nine contacts in the native form. For this reason, the almost native form is metastable. In order to get from the almost native form to the native form, individual HH contacts must be broken and new HH contacts formed. The refolding of these two forms of energy into one another progresses slowly, because the connecting intermediates are less stable and thereby lie higher up in the folding funnel than the two (meta)stable shapes. For the two stable forms, the HH contacts are compressed in an inner core of the lattice; by this means, contacts with the solvent are reduced. This is termed a hydrophobic effect, probably the biggest driving force of the protein folding.

*Aspects of complexity and/or complexity processes in protein folding – achievements of the HP lattice model*: from what has been said thus far, the exponential scaling of the calculation steps with increasing number of amino acids becomes understandable. It also becomes understandable that the type of interaction and their distance‐ and orientation‐dependence in real proteins is difficult, in some cases impossible, to describe. The interactions which a side chain *i* takes part in depend on the conformation of many other side chains. Hitherto, we have only been able to calculate these interaction terms approximately, by means of molecule dynamics simulations based on classic Newtonian force fields. The number of atoms to be dealt with is too large for quantum mechanics calculations, and, in particular, the interactions between protein and solvent are not adequately understood. On the other hand, the numerical effort for the calculation of the thermodynamic aspects of folding can be stated mathematically. In addition, the principal boundaries of enthalpic and entropic stability can probably be named at least approximately. These aspects, therefore, give rise to a description of the complex system of protein folding with respect to the number of elements (atoms and/or amino acids), the forces between these particles (Coulomb, Lennard‐Jones etc.), the number of possible states, the energy of these possible states, the external energy input into this system and also the behavior of the system under energy input. It is astonishing that folding in a system which exhibits all the features of a complex system still progresses correctly and reproducibly. The evolution of the complex system happens unambiguously for a gene in experimental conditions [102, 103]. In general, biochemical/biophysical experiments assume that biological function originates in ensembles of the same protein, i.e. that individual protein chains are not functional. The size of the ensemble here is in the order of magnitude of 10^5^–10^17^particles. In general, the ergodic assumption applies: the ensemble of the 10^5^ particles behaves in the way that an individual particle – observed over a long time – would behave, i.e. the mean value of a property of the ensemble is equal to the temporal mean value of the same property of the individual molecule [104]. In general, the function of a protein is coupled to the 3D conformation, if also frequently to weakly populated, activated states in which not all native contacts are formed, but which essentially retain the 3D conformation of the protein. Intrinsically disordered proteins are an exception to this concept. In evolutionary terms, the coupling of folding, of properties of amino acids and their functional diversity (variety) is by no means mandatory, but rather “unpredictably new”. In order to catalyze a chemical reaction, an enzyme must, within a 3D conformation, position individual reactive atoms in a certain spatial location to form the active side of the enzyme. In what 3D position this succeeds, and which atoms are best suited to exert catalytic function and that all biochemically necessary reactions for life can be catalyzed by enzymes is “unpredictably new”. Biomacromoleules must further exert their function in narrow temperature ranges in an aqueous environment. That the evolved molecules can do this is “unpredictably new”, since the properties of the atoms (including their complementary) are uncoupled from evolutionary cycles. In other words, whether the existing atoms with varying abundance could in fact be the building blocks of life “was” unpredictable, yet, since the evolution of chemical could not be reversed in a “new big bang”, the capabilities of atoms to lead to life was unpredictable.

*Time complexity of the protein folding problem*: In information technology, the number of computer operations required to solve a problem is an intuitive indicator of its complexity. Fundamental to this is the question of with what function the effort necessary for computing grows with the number of entries, i.e. with the size of the system to be computed. This consideration gives rise to the complexity of classes P and NP. A problem is in the P class if it is solvable in polynomial time, i.e. if the necessary computational effort grows in the form of a polynomial with the size of the system being investigated. Problems in P are seen as viable, because even if, as in the above example, the interaction contributions of each possible amino acid pair are to be approximated for a given conformation, the number of computer operations grows only with a function of the order of magnitude n^2^ (higher indices are also possible). In contrast to this are problems of the class NP, where the number of necessary computer operations grows exponentially with the number of entries, and a solution in a viable computation time is only possible for very small systems [105]. A classic example of this is a protein folding algorithm, which evaluates each possible conformation and therefore remains insolvably inefficient despite gross simplifications of the model. If e.g. each amino acid in a protein can adopt only two different conformations, then this generates, for a protein of 129 amino acids, 2^129^ = 6.8*10^38^ possible total conformations. Even a hypothetical supercomputer, which analyzes 10 trillion (10^13^) possible conformations per second, would require 6.8*10^38^ *10^−13^s = 6.8*10^25^s>10^20^ years for such a calculation. Our universe is only 13.7 billion (1.37*10^10^) years old. Problems in the NP class also have the interesting feature that, despite their complexity, a predetermined solution can be verified in polynomial time, i.e. efficiently. Calculation of the thermodynamic stability of such a protein conformation would be equivalent to such verification. Despite the numerous interactions, which here may have to be added together by way of simplification, such a calculation is efficient in the theoretical sense. If, however, an algorithm tries to find the conformation with the lowest energy, every conformation must be evaluated for this. An everyday example of this imbalance is Sudoku puzzles, in which finding a solution is complex, but where verifying a given proposed solution is simple, even for large systems. The encryption of data also makes use of this principle. Since every problem in P is also solvable in NP (P is therefore a subset of NP), we need another definition for particularly difficult, i.e. “pure” NP problems. We term these “NP‐complete”. From a theoretical perspective, protein folding is typically seen as an NP‐complete problem [106].

*Kinetic problem of protein folding*: Why is considering protein folding from an information technology perspective useful? Because the same problem exists in Nature and is solved there extremely efficiently. Similar to the computation example above, the astronomical number of possible states has, here too, been recognized as problematic. This is referred to as Levinthal's paradox of protein folding [107]. The time needed for an amino acid to change from one conformation into another depends on the activation energy for these changes. Even if one assumes very small activation energies, which are accompanied by very short times of τ∼10^−1^ ^3^s (10 trillion conversions per second), the time needed for 129 possible amino acids to adopt all conformations once and hence “to fold” a protein via stochastic means, is of the above‐named order of magnitude (10^20^ years), a timeframe that is longer than the age of the universe. Experimentally, however, one observes that proteins fold very rapidly, depending on the experimental conditions, within a few milliseconds to seconds.

From practical experience, the complexity classes P and NP are assumed as different from one another, although so far this has had no mathematical proof. What is referred to as the P‐NP problem is one of the fundamentally unsolved questions in mathematics. Levinthal's paradox and the most recent successes in protein structure prediction by means of *machine learning* could therefore provide an indication that potentially all problems of the NP class can be reduced to complexity P with the right algorithm and therefore efficiently solved, that therefore in reality it is true that: P = NP. It is more likely that protein folding, in reality, is not an NP‐complete problem, since polypeptide chains do not fold purely stochastically but along an energy landscape (Figure 15), in which only a vanishingly small proportion of the conformation space theoretically possible is actually populated. Levinthal himself proposed, as a solution to his imaginary experiment, that rapidly formed local interactions would act as nucleation cores for subsequent folding steps, which is why the term ‘paradox’ can probably be viewed as an exaggeration [106]. Indeed, a simple mathematical model demonstrated that even the introduction of minimal energetic “penalties” for unfavorable local interactions lowers the estimated folding time to an order of magnitude that is biologically relevant [108]. Because secondary structures and local contacts can be formed more quickly than non‐local (tertiary) interactions, a folding process heavily simplified by modularity can be assumed [109]. In addition, avoiding non‐productive, local energy minima plays a key role here. However, an energy landscape optimized for rapid folding is not a basic property of proteins as a category, rather, coded in the respective amino acid sequence after evolutionary optimization [110]. As shown in Table 2, 1.26*10^130^ different sequences are possible for what, with 100 amino acids, is a relatively small protein. In contrast to this, there are around 200 million (2*10^8^) natural protein sequences which have been detected or derived from genome analyses. This means that the theoretical sequence space, similar to the possible conformation space, in which reality can be assumed as almost empty, and that the tiny islands of existing sequences are probably optimized for extremely rare properties. The ability to fold rapidly and efficiently is probably such a property which could explain the discrepancy between theoretical and practical complexity of the protein folding problem. For a more in‐depth discussion of the topics of Levinthal and complexity, see Ngo et al. [111].

## 9 Kinetic Aspects of Cellular Information Transfer

While the “unsolvable” tag is no longer entirely accurate, the “protein folding problem” has evolved into a new phase focusing on speed, accuracy, and the complex dynamics of protein interaction in vivo. Cellular information transfer is the passing‐on of genetic information from a parent cell to a daughter cell and the conversion of this genetic information into function during transcription and translation. So far, we have discussed the respective synthesis steps for biopolymers and the subsequent folding of the 3D conformation of RNA, and protein. Such discussion is based on the work by Anfinsen, since the information about the 3D conformation is contained in the amino acid sequence. Recently, however, we have been able to show in experiments that the kinetics of transcription (RNA synthesis) and translation (protein synthesis) have a key influence on of the (transient) formation of functional relevance of RNA and protein structure. Thus, it is not sufficient to discuss complexity without RNA‐based regulation systems regulate transcription in a kinetic manner. In riboswitches, for example, regulation can be achieved only during RNA synthesis and is exerted by RNA transcription intermediates as opposed to full‐length RNAs. These transcription intermediates adopt metastable conformations; after transcription has ended, the full RNA transcript is no longer active in regulatory terms [112, 113, 114, 115, 116]. For RNA‐based regulation, these experiments and their theoretical quantitative modeling show a coupling of the kinetics of RNA synthesis, the simultaneous folding of the RNA into a functional structure, and structure‐dependent regulation.

We discussed the origin of the genetic code. It provides the translation specification for which base triplets code for which amino acid. Base triplets of four different RNA building blocks can code for up to 4^3^ = 64 different amino acids. We have seen that there is one start codon and three stop codons. Only the two amino acids tryptophan and methionine are coded for by a single codon, all other amino acids are coded for by up to six different codons, with the third RNA building block in particular being very variable. The use of these synonymous codons varies widely in different organisms (Table 3) [117].We have seen that there is one start codon and three stop codons. Only the two amino acids tryptophan and methionine are coded for by a single codon, all other amino acids are coded for by up to six different codons, with the third RNA building block in particular being very variable. The use of these synonymous codons varies widely in different organisms (Table 3) [117].

**TABLE 3:** Varied use of synonymous codons, taking the example of codon use for the amino acid arginine (Arg) in a bacterium *(Escherichia coli)* and in a cow *(Bos taurus)*.

Codons for the amino acid Arg	Use of the codons (absolute values) per thousand codons	
Small proteins	*E. coli*	*B. taurus*
CGG	7.9	12.5
CGC	14.0	11.1
CGA	4.8	6.4

Only recently were we able to contribute to the understanding, above all also of the functional consequences, of the use of different codons together with the groups of Komar and Rodnina [118, 119]. These investigations introduce a kinetic aspect to cellular information transfer, in particular for the folding of a protein while protein biosynthesis is taking place. The work showed that, depending on the precise speed of incorporation of each individual amino acid during the translation of the eye lens protein γB‐crystalline, the oxidation state of cysteine groups in the protein is changed, which results in different stabilities and local structures in the differently translated protein. Based on these observations, it is necessary to expand the description of cellular information transfer to include a kinetic dimension.

**FIGURE 16:**
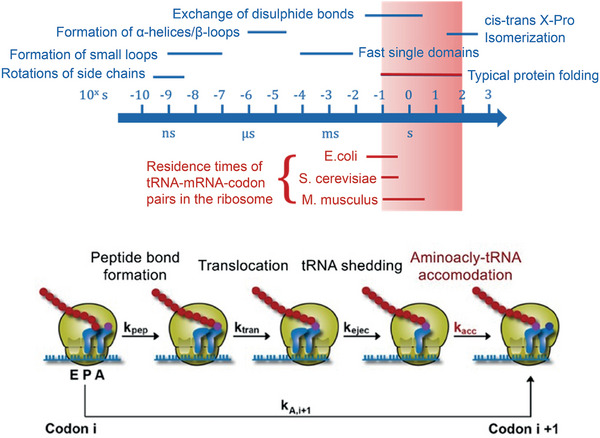
Time periods for folding processes and for protein biosynthesis. It becomes apparent that protein folding progresses on the same time scale as protein biosynthesis (timescale indicated in red). Reproduced from Buhr et al., Molecular Cell 61(3):341–351, 2016, https://doi.org/10.1016/j.molcel.2016.01.008, 2016 Elsevier Inc. Used with permission [118].

Figure 16 compares the timescales on which conformation changes occur within a protein with the speed of the protein translation. Processes relevant to folding take place on timescales spanning twelve orders of magnitude. Rotations around single bonds take place on a timescale of 10^−10^ ns, the folding of simple secondary structural elements such as, in particular, α‐helixes, requires 10^−6^ µs and the folding of proteins with a single domain can progress in as little as a millisecond (10^−3^). Typical folding times of proteins lie on a timescale from 100 ms to 10 s (10^−1^–10 s). On the other hand, one assumes the times spent on the ribosome by a codon‐anticodon pair made from mRNA and tRNA to likewise be between 100 ms and 1 s, with some variations between different organisms. The speeds of the folding and of the protein synthesis are therefore on the same timescale.

**FIGURE 17:**
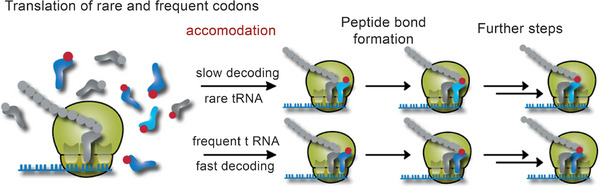
Translation of rarely and frequently used codons. The growing peptide chain is symbolized by the gray chain of a tRNA in the ribosome. On the right next to the binding site of the tRNA, what is known as the P site, is the A site in the ribosome. Here the next codon is located on the mRNA (blue chain at the lower end of the ribosome). The different tRNAs are all located outside of the ribosome. They are present with differing frequencies, so they have different concentrations. A frequent tRNA, symbolized here by the dark blue L‐shaped tube, results in rapid decoding of the mRNA, whilst a rare tRNA, symbolized here by the light‐gray L‐shaped tube, results in slow decoding. Only when the right complementary tRNA is bound at the A site of the ribosome is a new peptide bond formed.

During protein translation, in each elongation step one codon of mRNA is, in each case, accessible to its complementary anticodon from the corresponding tRNA. Here, the recruitment of the tRNA is the rate‐determining step and depends on the absolute and relative concentrations of the matching tRNA species. Here the correct codon‐anticodon interaction can be preceded by the trying‐out of several incorrect codon‐anticodon interactions due to the binding of an incorrect tRNA. mRNA codons with more frequent tRNAs have a greater chance of the correct codon‐anticodon interaction and are therefore, on average, read off faster than codons with rarer tRNAs (Figure 17). Because the respective tRNA concentrations correlate with the corresponding codon frequencies in the genome, the relative codon use in an organism may be used as an approximation for the translation speed (Table 3). The same amino acid can therefore be integrated with differing degrees of rapidity depending on which synonymous codon is used for the translation.

**FIGURE 18:**
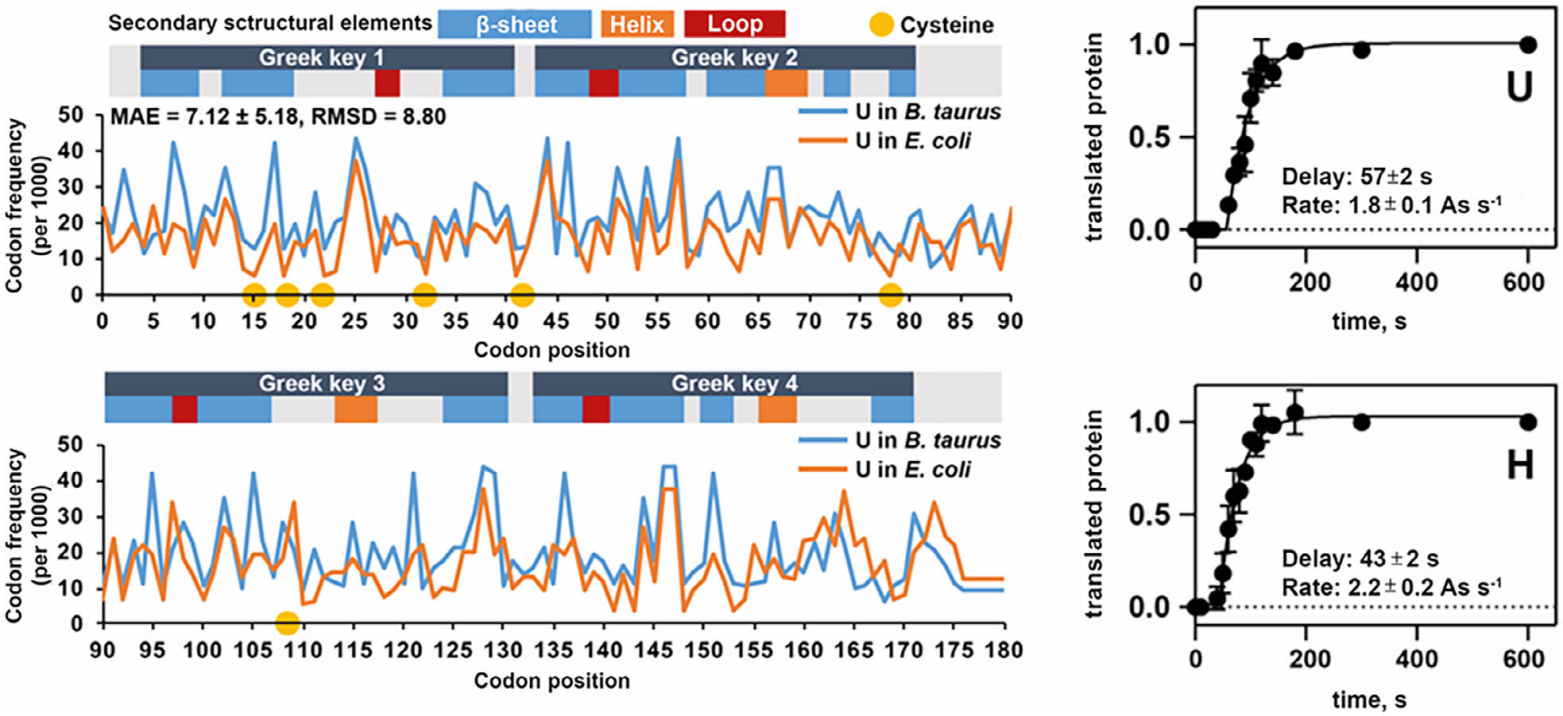
(left) Codon use of the genetic information of the model protein γB‐crystalline in the cow (*B. taurus*) and in a bacterium (*E. coli*). The position of cysteine side chains in the protein is marked by yellow circles, the secondary structural elements in the protein from two domains and are indicated in boxes; (right) speed of translation as a function of the mRNA sequence. U stands for the identical mRNA (unmodified) which from the cow is expressed heterologously in a bacterium, H stands for an mRNA in which the speed of translation between the two organisms has been adapted through the swapping of synonymous codons (harmonized). Reproduced from Buhr et al., Molecular Cell 61(3):341–351, 2016, https://doi.org/10.1016/j.molcel.2016.01.008, 2016 Elsevier Inc. Used with permission from Ref. [118].

Figure 18 shows the variation in the codon use of the same mRNA sequence of the protein γB‐crystalline during translation in a cow and a bacterium. Proteins do not fold only after their synthesis on the ribosome but also continue doing so within the cell after the release from ribosomes. Folding and unfolding of a protein therefore occur permanently. The detection of initial differences during the coupled translation and folding of the fully synthesized protein therefore requires that these differences be stored in some way during the first folding directly after synthesis. So far, there are three different possible mechanisms for this: the swapping of entire domains of the protein from the folded state, the shifting of secondary structures between different arrangements, in particular “β‐register shifts”, and differences in covalent modifications of the protein. For the model protein γB‐crystalline we were able to show that the oxidation states of cysteine amino acids of the protein differ depending on the synonymous mRNA sequence used.

**FIGURE 19:**
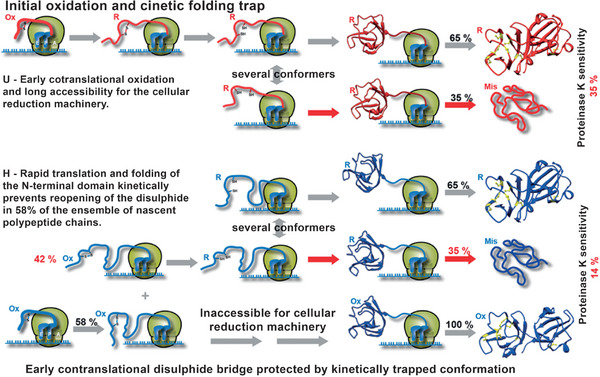
The model for the coupling between synthesis and folding of the protein γB‐crystalline using two synonymous mRNAs U and H. Reproduced from Buhr et al., Molecular Cell 61(3):341–351, 2016, https://doi.org/10.1016/j.molcel.2016.01.008, 2016 Elsevier Inc. Used with permission from Ref. [118]. The cytosol in the bacterium *E. coli* is, in principle, reducing. This leads to disulfide bridges between cysteine side chain being broken, except when, through the 3D conformation of the nascent polypeptide chain, the disulfide bridge is sterically shielded. For mRNA (H) the protein synthesis progresses faster than for mRNA (U). (Top): The protein synthesis using the codons of mRNA (U) is too slow for oxidized cysteine side chains to be sterically protected. Because the side chain disulfide bridges contribute an important function toward folding stability during the translation, only 65% of the protein can be correctly folded after translation, 35% aggregates (Mis). (Bottom): For mRNA (H) the protein synthesis is so fast that the entire N‐terminal domain of the two‐domain protein has already been generated and by this means the cysteine bridge which is already 58% generated protects against reduction. Folding in this domain does not result in aggregates. For the reduced form without a cysteine bridge (42%), one likewise observes 35% aggregation. In the case of translation of the mRNA (H), one isolates 58% folded protein with a disulfide bridge, 15% aggregated protein and 27% folded protein without a disulfide bridge. The formation, postulated by the model, of a disulfide bridge already in the exit tunnel of the ribosome has now been confirmed and it has thereby been proven that protein modifications can already occur in this tunnel. The data show that, astonishingly, both non‐native and native disulfide bridges are formed [119].

In addition, we were able to show that these modifications are heterogeneous and can already take place in the exit tunnel of the ribosome [119]. When jointly observing the speeds of the protein synthesis and folding, the following picture emerges: the synthesis and folding trajectories fan out for different protein molecules within the ensemble (Figure 19). Interestingly, the heterogeneity is also observed for γB‐crystalline isolated from cows’ eyes. Optimization of protein synthesis speed through evolutionary selection of synonymous codons, in conjunction with kinetic requirements for productive protein folding, results in altered protein folding trajectories during protein synthesis and reversible folding and unfolding processes in the cell and in vitro. During the translation, parts of the protein are released from the ribosome (represented by blue spheres).

**FIGURE 20:**
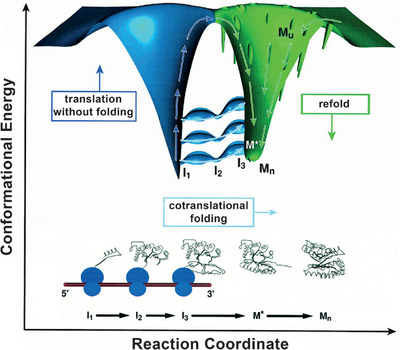
Graphic model to illustrate the coupling between protein synthesis (translation) and co‐translational folding. Reproduced from Buhr et al. as per the publication by Fedorov and Baldwin, Molecular Cell 61(3):341–351, 2016, https://doi.org/10.1016/j.molcel.2016.01.008, 2016 Elsevier Inc. Used with permission from Refs. [118, 121]. The protein chain becomes even longer through its synthesis; by this means the folding landscapes are continuously changed. Whereas a fully synthesized protein chain must overcome high barriers of intermediates I_1_, I_2_, I_3_ to reach the next state M, these high barriers in terms of energy can be avoided in the case of co‐translational folding.

These parts, as sub‐elements, can already fold themselves. Employing sub‐element folding, they thereby sidestep Levinthal's paradox of the astronomically large conformation space through the restriction to local interactions. In this scenario, vectorial folding may occur, i.e. the N‐terminal domain of a protein may fold first. Remarkably, Levinthal himself already considered this possibility [120]. Through the insertion of rare codons and thereby slowly translated proteins, this process can be facilitated further. The point of overlap of the blue and green funnels indicates the point at which the entire protein has been synthesized and the kinetics of folding are identical to the folding kinetics after release from the ribosome (refolding). The left‐hand side of the blue funnel symbolizes the growing protein chain, which from a certain threshold length forms stable secondary structural elements and hence can adopt a stable conformation. The waves connecting the blue and green funnels symbolize accelerated co‐translational folding trajectories (Figure 20).

**FIGURE 21:**
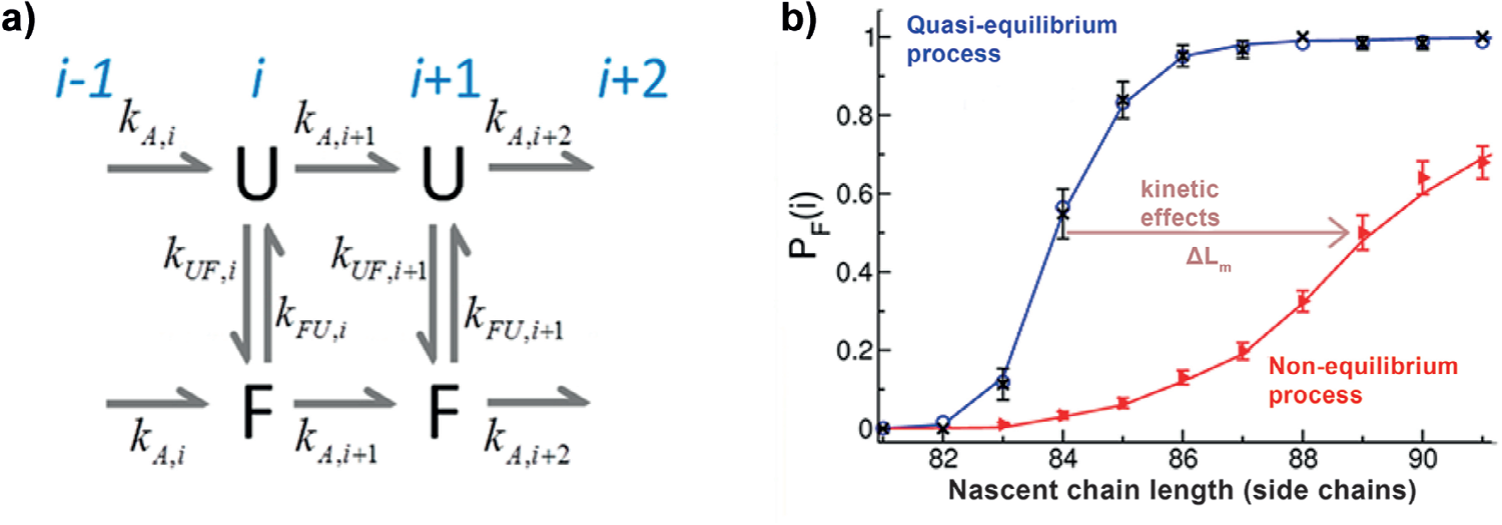
Modeling of the kinetics of protein synthesis and folding for two different extreme scenarios. Reproduced from Buhr et al., modified from O'Brien et al., Molecular Cell 61(3):341–351, 2016, https://doi.org/10.1016/j.molcel.2016.01.008, 2016 Elsevier Inc. Used with permission from Refs. [118, 122].

These aspects can also be quantitatively modeled (Figure 21). Here, the elongation rate of a growing polypeptide chain on the ribosome (characterized by the rate k_A,i_) and the kinetics of their folding k_UF,i_ are the key parameters (Figure 21a). A distinction can be drawn between two cases: 1. If the synthesis rate is slower than the rate of protein folding (k_A,i_ < k_UF,i_), then the folding is in equilibrium and is driven thermodynamically. If k_A,i_ ∼ k_UF,i_ or k_A,i_ > k_UF,i_, then the nascent chain does not have sufficient time to fold before the length of the chain outside of the tunnel changes again. Then, this chain is in a state of non‐equilibrium and kinetic effects may have an impact on the final folded state. This is the reason for kinetic aspects of cellular information transfer.

## 10 Classification of the Role of Chemistry in the Research Field “Complexity”

In this review, the evolution of life has been outlined insofar as methods from chemistry are relevant to this. To conclude we would like, where necessary and not self‐evident, to compare the aspects presented with the general definition of the evolution of complex systems in Section 2.

1. A dynamically complex system evolves over time. In the strict sense, its evolution is irreversible and not reproducible.

These aspects are extensively applicable in systems between prebiotic chemistry and the evolution of the first living systems. The special aspect of irreversibility exists especially regarding the astronomically large sequence space which opens during the polymerization of four different nucleotides or twenty different amino acids. The astronomically large sequence space does not permit full representation of all possible sequences. Instead, initially formed sequences accumulate as opposed to all other sequences, even with just a small selection advantage. The accumulating sequences are therefore a subset of all possible sequences. Point 1 negates the possibility that, if everything was put back to the beginning, evolution could progress again in the same way. At this point, starting with Jacques Monod's book title “Chance and Necessity” one begins to reflect: could, because of the universal applicability of natural laws, only those atoms emerge which did emerge? Could, with this reservoir of atoms, only those prebiotic molecules develop whose etiology is outlined by Albert Eschenmoser? Arguments in favor of this are the extraterrestrial findings. Could, from these prebiotic molecules, only those develop which we find in existence today? Does chance then come into play in the expansion of the possible sequence space, which is necessarily generated through the polymerization of monomeric RNA and DNA building blocks [123]? Then, the property of polymerization would be necessary in the sense of mandatory, likewise self‐organization and autocatalytic self‐replication. And life would begin in the opening‐up of a sequence space in which random individual molecules are selected and these then initially amplified by means of autocatalysis. Put simply: life begins where, through chance, a selection is made from an infinite number of possible structures, but this selection has binding consequences. This selection is historic. Repetition of evolution, the emergence of a new world, would produce the same elements (atoms, molecules, polymers) but these would not be linked in the same way.

2. Complex systems are open systems. This implies that, in principle, separation of the complex system from its environment is possible. An unavoidable exchange exists in the form of energy. The complexity of the environment may frequently be greater than the system complexity.

Point 2 of the definition of complex systems becomes important, for Eigen's system of the permeable box. The permeable box makes it possible to draw up differential equations for the mathematical description of a primitive cell, which separates inside and outside, and partitions material transport and the energy input associated with this material transport. Without this separation, the transition to life fails, because then the dilution factor V_i_ (Equation 3) becomes dominant. The exchange between the inside and outside in the permeable box constitutes an initial system of anabolism and catabolism. The permeable box makes it possible for the system, in terms of energy input, to first be open, and second also to not be able to escape the energy input. A characteristic feature of the system at the threshold between the animate and inanimate is that complexity is generated within the system. Initially randomly emerging information reproduces thanks to autocatalytic self‐replication. In autocatalytic self‐replication, the term “random” means that the exact sequence of the two RNA strands – RNA template and RNA primer – is random; they only need to be self‐complementary (Figure 9). Autocatalysis already begins with such RNA templates and RNA primers which could still emerge through stochastic polymerization. Precisely in the transition between the random RNA sequences and those capable of self‐organization, information arises through autocatalysis (selection of a specific RNA sequence over other possible RNA sequences) which is retained in the system and passed on. One could speak here of conserved information, thereby making a specific distinction between a random sequence of RNA building blocks and information that is conserved because it is enhanced through self‐replication and amplification.

Is the complex system always less complex than the world around it, asks Niklas Luhmann? From our perspective, this question cannot be answered unequivocally and touches different levels. The world outside of the permeable box is not subject to any self‐restriction or any selection. The reduction of the astronomical sequence space and the accompanying selection did not occur outside of the permeable box, the world of the possible is not limited, the complexity potential not restricted (however, see here also the discussion regarding Point 1 on necessities) [124, 125]. On the other hand, the restriction of the complexity potential is a prerequisite for the evolution of new functions, described in the transition between an “RNA‐only world” and an “RNA‐protein world”, which emerges through hypercycles. In addition, the complexities, in principle, of the world inside and outside are not fundamentally different, rather, the probabilities of occurrence of a certain event from the astronomically large number of all possible events are extremely different.

3. The configuration of a system is describable at any point in its evolution, and possible at any point within the laws of the system.

This describability in principle exists analytically speaking at any point in the evolution of the system. Distinct from this is the predictability, which is not possible, both because of the excessively large sequence space and, in principle, because of the stochastic fluctuations of the system.

4. The elements of a complex system may be arranged in different hierarchical orders. In general, there is an exchange between these hierarchical levels; this exchange may be reversible or irreversible, directional or in equilibrium. Frequently, but not necessarily, complexity evolves in dynamically complex systems at higher levels from the characteristics of the elements of lower levels. In time‐independent, complex systems, complexity may be a static characteristic of higher orders.

Eigen's example of the evolution of hypercycles, in which the self‐replication of the RNA plus the chemical link between two different kinds of polymers (RNA and proteins) passes on the blueprint for proteins and therefore the polymerizations of these different functional polymers are coupled, produces a functional advantage over the more primitive, simple cycle. The hypercycle describes the emergence of hierarchical orders. This hypercycle functions only through the clear coupling of the cycles in the interests of the shared information, and in the case of RNA and proteins is directed from the information‐carrying RNAs toward the function‐carrying proteins, in other words, in terms of information transfer it is directional and irreversible. This directional information flow is not reversible in the sense of information being transferred from the protein to the RNA. The hypercycle is an example of how complexity at a higher level develops from the elements of lower levels. The hypercycle first works by the RNA sequence, which codes for a protein that catalyzes RNA replication, having a selection advantage. RNA does not lose the role of information template (the negative copy as defined by Haldane, see reference 35). During the hypercycle, however, proteins may then be generated that react to a different selection pressure. By this means, a new selection pressure is generated, which is responded to in the second level of the hypercycle. Selection takes place by selecting functional properties of proteins. The replication of the information‐carrying RNA molecules must, for this, necessarily be inherently error‐prone, so that new mutants can result in new proteins that have a functional advantage. This point is remarkable and breathtakingly exciting: For the evolving system, the level changes at which selection pressure is exerted. In the case of the evolution of life, in the “RNA‐only world” a selection pressure initially acts on those RNA sequences, which can self‐replicate most efficiently. In the transition to the primitive “RNA‐protein world”, those particular RNA molecules are selected that carry amino acids and therefore provide a reason for the informed construction of polypeptides (unlike the random polymerization of polypeptides). How a selection pressure for short peptides could already be generated here remains unclear, since these short peptides must already exhibit a functional selection advantage.

If, however, sufficiently long proteins have then emerged, these proteins take over autocatalysis and the error checking of the self‐replication of the RNA system. From this point of takeover, the site of action for selection shifts. In this sense, the “RNA‐protein world” is more complex than the “RNA‐only world”, since the selection, and linked to this the regulation, becomes more indirect and has more starting points. Properties of proteins and of their changes caused by mutations may result in a selection advantage, as the synthesis specifications of the two categories of polymers are coupled. Whilst in the RNA world, sequence and property are linked, and genotype and phenotype can be found in a polymer, in the “RNA‐protein world” these functions are split up and are performed by the RNAs or proteins. The transition from the “RNA‐only world” to the “RNA‐protein” world is one of the key emergence points in the evolution of life.

5. The evolution of a complex system is, in principle, driven by an almost always constant energy input. The effect of this is that systems pass from a state of lower complexity to a state of higher complexity. The extent of the complexity is describable and/or quantifiable within the theory of complex systems.

Consistent with Stegmüller, it has been shown that Eigen's theory of the evolution of matter permits precisely this quantifiability and is in accordance with the second fundamental law of thermodynamics.

6. In the transition of both static and dynamic complex systems at points or in periods of criticality, complex systems become emergent. This emergence may exist on a transition of all or only individual elements of the system.

In the outlined history of the evolution of living systems, there are many emergence points. The development of prebiotic, complementary, polymerizable compounds from the simple atoms and molecules that developed in stellar space is a point of emergence. The development of complementary compounds permits the self‐organization of matter. The transition from self‐organization to self‐replication is a second point of emergence. The coupling of two different categories of polymer, RNA and protein, into a hybrid molecule (tRNA) and the origin of the genetic code is a further emergence point. The folding of proteins, extending as far as highly functional biomacromolecules, and the coding of kinetic information as a part of function is a last example of the emergence points discussed here. It should also be remembered here, however, that emergence and complexity are two separate phenomena. Emergence does not constitute complexity. Particularly on the points, outlined here, of the transition from the “RNA‐only” world to the “RNA‐protein” world, the chronologically later system stands out for a new type of complexity characterized by the change in the selection. The “RNA‐protein” world is not reducible to its precursor; and the two worlds cannot be represented on a continuous complexity scale. The “RNA‐only world” is inherently complex, like the world of potentially prebiotic molecules is complex and not reducible from the “RNA‐only” world.

## 11 Challenge and Future Perspectives

Is the research into complexity and emergence in chemistry completed? Especially in Section 9, we have presented aspects of a new level of complexity regarding the coupling of information transfer between the information to be transferred and the speed, therefore the kinetic modulation, of the information transfer. Biochemical and structural biological research into these kinetic aspects is the subject of our natural science research. The integration of the implications of the kinetics of complexity into a transdisciplinary research program is of further intensive discourse as part of modern natural philosophy.

With the development of artificial intelligence and its massive use, transdisciplinary research in complexity and emergence will become increasingly difficult. The approach to try to conduct a coherent, time‐independent and reproducible model building, intended within this review, might be replaced by a descriptive model that does not delineate the requirements of a complex system (as opposed to a complicated system) and the requirements of an emergence and that becomes fluctuating as the basis of knowledge recovered by AI‐based search algorithms is constantly changing.

It is a view of natural sciences that understanding of a living system requires its faithful reconstruction. This explicit statement of this principle is linked to Feynman's statement “What I cannot create, I do not understand”. If not mistaken, it is the overall vision of Systems Biology to move from collecting descriptive data of living systems to generating new living systems. This novel art of data collection is at the heart of research programs that aim at developing a digital twin of the cells, for example.

Based on the discussion given within this review, emergence of new systems, while being describable within the framework of natural science, in particular physics, is fundamentally unpredictable, and thus can also not be constructed by mankind. Thus, this acknowledgement of failing remains at the core of reflecting on complexity and emergence.

We further believe that there is a necessity to increase our understanding of complexity in a transdisciplinary manner as the development of societal trends are endangered at multiple levels, including abandonment of international scientific exchange, fact‐based decision‐making, and scientific lead in the generated precise data for mega trends. Part of the success of populistic current trends is its explicit statement against complexity, discrediting the necessity of (transdisciplinary) research that aims to cope with complexity. Adaptation of a “splendid‐isolation” approach or denial of an important role of Chemistry in the understanding of complexity and emergence belittles the impact of Chemistry‐based models in contributing to solving the challenges of our current society.

## Conflicts of Interest

The authors declare no conflicts of interest.

## Data Availability

Research data are not shared.
